# Large‐displacement, hydrothermal frictional properties of DFDP‐1 fault rocks, Alpine Fault, New Zealand: Implications for deep rupture propagation

**DOI:** 10.1002/2015JB012593

**Published:** 2016-02-18

**Authors:** A. R. Niemeijer, C. Boulton, V. G. Toy, J. Townend, R. Sutherland

**Affiliations:** ^1^ Faculty of Geosciences, HPT LaboratoryUtrecht UniversityUtrechtNetherlands; ^2^ Geology and Geophysics, School of Environmental SciencesUniversity of LiverpoolLiverpoolUK; ^3^ Department of GeologyUniversity of OtagoDunedinNew Zealand; ^4^ School of Geography, Environment and Earth SciencesVictoria University of WellingtonWellingtonNew Zealand; ^5^ GNS ScienceLower HuttNew Zealand

**Keywords:** friction, earthquakes, hydrothermal, Alpine Fault

## Abstract

The Alpine Fault, New Zealand, is a major plate‐bounding fault that accommodates 65–75% of the total relative motion between the Australian and Pacific plates. Here we present data on the hydrothermal frictional properties of Alpine Fault rocks that surround the principal slip zones (PSZ) of the Alpine Fault and those comprising the PSZ itself. The samples were retrieved from relatively shallow depths during phase 1 of the Deep Fault Drilling Project (DFDP‐1) at Gaunt Creek. Simulated fault gouges were sheared at temperatures of 25, 150, 300, 450, and 600°C in order to determine the friction coefficient as well as the velocity dependence of friction. Friction remains more or less constant with changes in temperature, but a transition from velocity‐strengthening behavior to velocity‐weakening behavior occurs at a temperature of *T* = 150°C. The transition depends on the absolute value of sliding velocity as well as temperature, with the velocity‐weakening region restricted to higher velocity for higher temperatures. Friction was substantially lower for low‐velocity shearing (*V* < 0.3 µm/s) at 600°C, but no transition to normal stress independence was observed. In the framework of rate‐and‐state friction, earthquake nucleation is most likely at an intermediate temperature of *T* = 300°C. The velocity‐strengthening nature of the Alpine Fault rocks at higher temperatures may pose a barrier for rupture propagation to deeper levels, limiting the possible depth extent of large earthquakes. Our results highlight the importance of strain rate in controlling frictional behavior under conditions spanning the classical brittle‐plastic transition for quartzofeldspathic compositions.

## Key Points

- Alpine Fault rocks are most frictionally unstable at 300°C
- Velocity dependence of friction depends strongly on velocity and temperature
- Alpine Fault rocks are frictionally weak at low velocity and temperatures less than 300°C

## 1 Introduction

**Figure 1:**
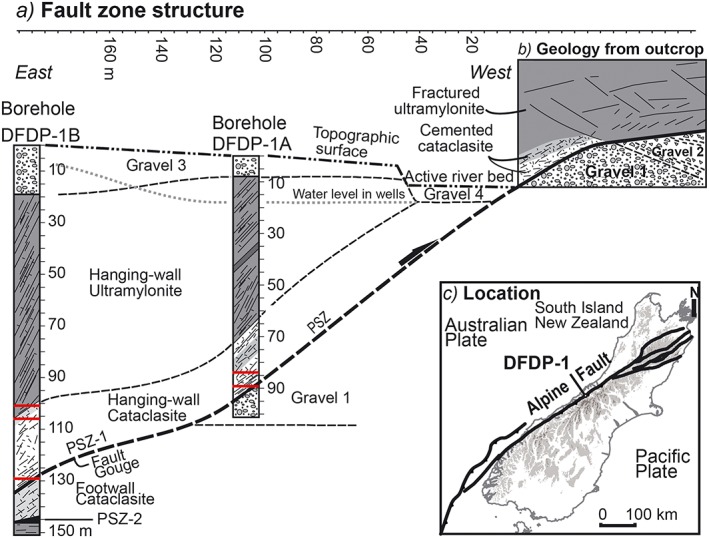
Location map and materials used in friction experiments. (a) Lithological cross sections of the two boreholes drilled during phase 1 of the Deep Fault Drilling Project (DFDP‐1), Alpine Fault, at Gaunt Creek. Horizontal axis (distance between boreholes) located across the top of figure. Samples used in this study were collected from the depths indicated by red lines. (b) A schematic cross section of prominent fault scarp outcrop geology on the south side of Gaunt Creek. (c) The Australian‐Pacific plate boundary in the South Island of New Zealand. Figure and caption modified from *Sutherland et al*. [2012].

The Alpine Fault is a major plate‐bounding fault that has accumulated over 460 km of dextral offset since 45 Ma, between the Australian and Pacific plates [e.g., *Sutherland et al*., 2000]. Paleoseismic evidence and the absence of measurable, historic surface deformation, i.e., surface creep, indicates that the Alpine Fault slips in large magnitude (*M_w_* ~ 8) earthquakes with a recurrence interval of 200–400 years, the last of which occurred in 1717 Common Era [*Sutherland et al*., 2007; *Berryman et al*., 2012]. Along the central Alpine Fault, oblique‐reverse slip has uplifted the Southern Alps and exhumed fault rocks from ~ 35 km depth over the last 5 million years [e.g. *Norris and Cooper*, 2001; *Little et al*., 2005]. In the Late Quaternary, dextral slip at 25–30 mm/yr and vertical slip at 5–10 mm/yr has translated and uplifted a ductile fault rock sequence overprinted by both brittle structures and hydrothermal alteration products [*Norris and Cooper*, 2007; *Toy et al*., 2012]. To investigate the structure and hydrological properties of the crustal‐scale Alpine Fault, phase one of the Deep Fault Drilling Project (DFDP) drilled two boreholes at Gaunt Creek in 2011 [*Townend et al*., 2009; *Sutherland et al*., 2012]. During this phase, a principal slip zone (PSZ) gouge that defines the boundary between cataclasite and Quaternary gravel was cored in hole DFDP‐1A, and two PSZ gouges were cored within cataclasites in hole DFDP‐1B (Figure 1). In this paper we present results of laboratory experiments on these fault rocks. We applied pressures, temperatures, and strain rates that are comparable to those that exist at depths within the seismogenic zone of the Alpine Fault.

Observations of microseismicity indicate that the seismogenic zone of the central Alpine Fault spans a depth range of 3–10 km [*Boese et al*., 2012; *Leitner et al*., 2001; *Bourguignon et al*., 2015]. Rapid exhumation of amphibolite‐facies metamorphic rocks has resulted in high geothermal gradients along the central Alpine Fault (63°C/km) [*Sutherland et al*., 2012; see also *Koons*, 1987]. The high geothermal gradients measured at shallow levels in the crust are unlikely to persist to large depths [*Craw*, 1997], and geological estimates yield a more conservative depth‐averaged geothermal gradient of 40°C/km [*Toy et al*., 2010]. The deep transition from seismic to aseismic slip is probably linked to the onset of plasticity, which in quartz occurs at temperatures of 300–350°C [*Tullis and Yund*, 1977, 1980; *Scholz*, 2002]. Rheologically, this puts the seismic‐aseismic transition at a depth of about 8 km, which is roughly consistent with patterns of microseismicity [*Boese et al*., 2012].

Rate‐and‐state friction (RSF) theory stipulates that the occurrence of slip instabilities (i.e., earthquakes) can be linked with the velocity dependence of friction [*Dieterich*, 1979; *Ruina*, 1983; *Scholz*, 2002]. The empirical set of equations, known as rate‐and‐state friction (RSF) equations [*Dieterich*, 1979; *Ruina*, 1983] is given by

(1) $$\mu =\mu_{0}+a\ln\left(\frac{V}{V_{0}}\right)+b\ln\left(V_{0}\theta /d_{c}\right)$$

(2a) $$\frac{d\theta }{dt}=1-\frac{\theta V}{d_{c}}$$

or

(2b) $$\frac{d\theta }{dt}=\frac{V\theta }{d_{c}}\ln\left(\frac{V\theta }{d_{c}}\right)$$

where *μ* is the coefficient of friction (*μ* = *τ*/*σ*_n_′, where *τ* is the shear stress and σ*_n_'* is the normal stress minus pore fluid pressure, *P_p_*), *μ*_0_ is the coefficient of friction at a reference velocity, *V*_0_, *a* is a constitutive parameter characterizing the direct effect of *μ* related to an abrupt change in *V*, *V* is the current velocity, and *b* is a parameter characterizing the evolution effect of *μ* over *d_c_*, a characteristic or critical displacement. *θ* is a state variable that evolves according to equation (2a) (“Dieterich or aging law”) or equation (2b) (“Ruina or slip law”). In our inversions, we have used the Dieterich formulation, but either formulation yields the same results for the values of (*a‐b*). Laboratory experiments conducted on fault rocks under in situ crustal conditions can yield data on the seismogenic potential of crustal faults, typically in the form of the RSF parameters in equations (1) and (2). The RSF equations, when linked to the equations of motion of a spring‐slider analogue, can account for recurrent unstable slip events as well as transitional oscillatory slip and stable sliding [*Ruina*, 1983; *Gu*, 1984]. When applied to the kilometer‐scale of fault zones, models incorporating RSF equations can reproduce several important seismological observations including earthquake nucleation and rupture [*Rubin and Ampuero*, 2005; *Bizzarri*, 2011], earthquake afterslip [e.g., *Perfettini and Avouac*, 2004, 2007; *Perfettini and Ampuero*, 2008], and aftershock duration [*Dieterich*, 1992].

Previous experimental work has shown that gouges derived from Alpine Fault rocks from DFDP‐1A and 1B are frictionally strong, with a coefficient of friction *μ* of 0.6–0.8 and strengthen with increasing temperature [*Boulton et al*., 2012, 2014; *Ikari et al*., 2014, 2015]. Additionally, frictional sliding transitions from velocity strengthening to velocity weakening with increasing temperature due to changes in the RSF parameters *a* and *b* [*Boulton et al*., 2014; *Ikari et al*., 2015], similar to previous work on quartz and Westerly Granite. [*Blanpied et al*., 1995, 1998; *Chester and Higgs*, 1992]. However, the maximum temperature explored in the studies of *Boulton et al*. [2014] and *Ikari et al*. [2015] was limited to 350°C and 160°C (for wet conditions), respectively, and therefore a temperature‐induced transition back to velocity strengthening was not encountered. Here we present results from an experimental study of Alpine Fault rocks aimed at expanding the previously tested range of temperatures and sliding velocities to determine the relationship between the velocity dependence of friction and temperature for a set of five samples obtained during phase 1 of the Deep Fault Drilling Project (DFDP‐1), Alpine Fault. These samples have undergone rapid exhumation along the Alpine Fault and are representative of rocks that have deformed at crustal depths spanning the brittle‐plastic transition. Ultramylonites within the sequence have accommodated large ductile shear strains, whereas fault gouges within the principal slip zone reflect brittle deformation processes (see *Toy et al*. [2015] for details). During exhumation, the fault rocks have been altered by pervasive fluid flow and precipitation of authigenic calcite and, locally, smectite [*Boulton et al*., 2012; *Menzies et al*., 2014]. The alterations hinder our ability to infer the frictional properties of the Alpine Fault at depth, unless we can document how mineralogical changes influence frictional behavior. In this study we show that the frictional strength varies little between the five samples studied; we also show that the values of (*a‐b*) change more with changing temperature and sliding velocity than with small changes in mineralogy.

In the following, we present the experimental methods and results, before discussing the variability of friction with temperature for all samples, comparing the results with previous studies. We go on to discuss the velocity dependence of friction and try to identify the deformation mechanisms that control this. Finally, we discuss possible implications of our results for the brittle‐plastic transition on the Alpine Fault and the deep extent of seismicity.

## 2 Sample Description

Core samples obtained in phase 1 of DFDP have been described in detail by *Toy et al*. [2015] and *Boulton et al*. [2014]. Our samples cover a modest but representative range of the fault rocks obtained with two ultramylonite samples (AF5 and AF6), two cataclasite samples (AF1 and AF2), and one gouge sample (AF4) from the upper Principal Slip Zone of DFDP‐1B, which is inferred to be an active PSZ hosted entirely within cataclasite [*Sutherland et al*., 2012]. The borehole depth and mineralogy of our samples is given in Table 1. The mineralogy was determined using a Bruker Kappa Apex II diffractometer on bulk powders (grain size < 50 µm). Quantitative data was obtained using pattern matching and model curving fitting using the Bruker DIFFRAC.SUITE software. However, since we did not perform the full methods required to obtain reliable percentages down to ±5 wt %, we only report qualitative data here. The fault rocks consist of up to 80 wt % framework silicates and up to 10 wt % calcite, with the remainder phyllosilicate minerals. Only the PSZ sample contains a moderate amount of clays (namely, ~15 wt % smectite). The PSZ sample used in our experiments contains less smectite than the PSZ gouge used by *Boulton et al*. [2014], which was 26 wt %. This difference may result from sample variability along the core, different X‐ray diffraction (XRD) analysis techniques, or sample preparation.

**Table 1:** List of Samples Used in This Study and Their Mineralogy According to Semiquantitative XRD

Sample	Depth (m)	Hole	Description^a^	Composition
AF 1	83.70–83.80	1A	Unit 4—upper foliated cataclasite	Major: quartz
				Moderate: albite
				Minor: chlorite and calcite
AF 2	89.70–89.76	1A	Unit 3—upper unfoliated cataclasite	Major: quartz
				Moderate: microcline ands sanidine
				Minor: phengite, chlorite, and calcite
AF 4	128.1	1B	PSZ	Major: none
				Moderate: quartz, labradorite, oligoclase, and smectite
				Minor: none
AF 5	106.7	1B	Unit 2–4 mixture	Major: albite and quartz
				Moderate: chlorite
				Minor: phlogopite and calcite
AF 6	101.24	1B	Unit 2—brown‐green‐black ultramylonite	Major: oligoclase and quartz
				Moderate: chlorite
				Minor: calcite and phlogopite

a From *Toy et al*. [2015]. Major indicates > 30 w t%, moderate 10–30 wt %, and minor < 10 wt %.

## 3 Methods

### 3.1 Experimental Apparatus

We performed sliding experiments using the hydrothermal ring shear apparatus described in detail by *Niemeijer et al*. [2008] (supporting information Figures S1 and S2). Sample preparation involved disaggregating and crushing each fault rock using a pestle and mortar and sieving the powder to obtain a grain size fraction <125 µm. All material was used and clasts were not separated. Before each experiment, 0.5–0.6 g of sieved powder was evenly deposited (resulting in a ~1.2 mm thick layer) on the bottom Ni alloy (René‐41) piston equipped with Ni alloy inner and outer rings coated with a low friction Molykote spray coating (supporting information Figures S2a and S2b). The piston set was coupled using a retaining screw (supporting information Figure S2a), attached to the top piston head and lowered into a key lock in the bottom of the deionized water‐filled vessel. Once the vessel was closed with the upper nut, pore fluid tubes and water cooling lines were attached.

The entire vessel was placed inside an Instron 1045 loading frame. The top piston locks into a stainless steel cross bar equipped with two load cells with an individual range of 1.2 kN and resolution of 0.00012 kN (= 60 MPa and 0.006 MPa in shear stress). The cross bar halts the rotation that is applied to the pressure vessel (and thus to the simulated fault gouge) by a 170 W DC electromotor (Kern P310) in line with two or three 1:100 gear boxes. The electromotor is controlled via the analogue output of the 16 bit analog to digital (A/D) converter, which in turn is controlled by a LabView virtual instrument, allowing control of sliding velocity to within ~0.005 µm/s (for the case of two gear boxes).

Normal load, which was applied to the sample via the pressure‐compensated top piston head using the Instron frame, is measured and controlled using a 100 kN load cell, giving control to generally within 0.05 MPa on the 28/22 mm annulus of the gouge layer. The pressure‐compensating mechanism means that any applied normal load is transferred directly to the sample, regardless of the level of the fluid pressure (minus any load supported by the internal seals). After applying the desired normal load, nominal fluid pressure (typically 0.5 MPa) was applied using the manual high‐pressure pump (5 ml volume). Temperature was then increased using an internal 3 kW furnace. The desired test temperature was typically reached within 15–20 min after which the sample was left to equilibrate for between 15 min and 2 h, depending on the operating temperature.

Methods used to measure experimental data are outlined briefly below. Fluid pressure is measured using a digital pressure transducer with a full scale of 400 MPa and a resolution of ~0.015 MPa. Temperature is measured close to the sample through a K‐type thermocouple inserted in a dedicated hole in the bottom piston. The furnace output is controlled with a Eurotherm 2416 proportional integral differential controller using the temperature measured on the furnace element. Displacement normal to the simulated gouge layer (i.e., compaction/dilatation) is measured using two linear voltage differential transducers, one with a 100 mm range (resolution 0.5 µm) mounted on the bottom of the Instron drive and one with a 1 mm full scale (resolution 0.1 µm) mounted on the side of the Instron. Shear displacement is measured using a potentiometer mounted on the baseplate supporting the pressure vessel. One rotation of the vessel (and thus the lower piston) corresponds to 10 rotations on the potentiometer, giving a resolution in shear displacement of ~0.27 µm. Data from all channels are acquired using a 16 bit A/D converter at a rate of 300 Hz. The acquired data are averaged and recorded at variable rates between 10 and 300 Hz, depending on the sliding velocity.

### 3.2 Data Analysis

Acquired raw data forces were processed to obtain shear stress and normal stress data. The externally measured torque was corrected for fluid pressure and shear displacement‐dependent friction of the Teflon‐coated O‐ring seals using calibration values obtained in runs with a dummy sample of carbon‐coated PolyEtherEtherKeton with a known sliding friction; seal friction is typically around 0.15 kN (equivalent to ~7 MPa shear stress). Additional calibration runs have shown that the contribution of the Molykote‐coated confining rings to the measured friction is negligible under the current conditions, and we have thus not corrected for this [see also *den Hartog et al.*, 2012a]. The applied normal stress was corrected for the stress supported by the internal seals, the level of which was clearly visible during initial loading and was generally around 0.5 kN (equivalent to ~2 MPa normal stress acting on the sample).

The RSF equations (1 and 2a and 2b) were coupled with an equation describing the time evolution of slip velocity as a result of the elasticity of the system:

(3) $$d\mu /dt=K\left(V-V_{lp}\right)$$

where *K* is the stiffness of the system surrounding the actively shearing fault and *V*_lp_ is the load point velocity. The set of equations were solved simultaneously using a fifth order Runge‐Kutta method. The values for *a*, *b*, and *d_c_* were then obtained as the solutions of a nonlinear inverse problem using an iterative least squares method [see, e.g., *Reinen and Weeks*, 1993; *Blanpied et al*., 1998; *Ikari et al*., 2009].

### 3.3 Procedure

All experiments can be subdivided into four stages. In stage 1, we sheared the gouges for a displacement, *x*, of 10 mm at a sliding velocity, *V*, of 10 µm/s (“regular tests”) or 3 µm/s (“slow tests”), which was followed by a series of velocity steps. The velocity step sequences employed were 0.3‐1‐3‐10‐30‐100‐300 µm/s for the regular experiments and 0.003‐0.01‐0.03‐0.1‐0.3‐1‐3 µm/s for the slow experiments (Table 2). In most experiments, effective normal stress was *σ_n_*′=90 MPa and the fluid pressure was *P_p_* = 60 MPa in stage 1 (giving a total normal stress of *σ_n_'* = 150 MPa and a pore fluid factor, *λ* of 0.4). In the experiments performed at 600°C, we observed large temperature fluctuations (up to 50°C), presumably due to phase transitions in the fluid and/or convection. To stabilize the temperature, we employed a higher pore fluid pressure of *P_p_* = 120 MPa in these 600°C experiments, giving a total normal stress of *σ_n_'* = 210 MPa and pore fluid factor *λ* = 0.57 for stage 1. All slow tests were terminated after stage 1.

**Table 2:** List of the Experimental Conditions^a^

Experiment	Sample	*T* (°C)	*σ*_n_ b (MPa)	*P*_f_ (MPa)	*x* (mm)	*γ* (−)	*h*_0_ (µm)	*h*_f_ (µm)	Notes^c^
u106	AF 2	300	90‐120‐150	60/80/100	54.4	99.9	730	445	ss
u107	AF 1	300	90‐120‐105	60/80/70	54.1	79.2	856	620	ss
u110	AF 1	20	90‐120‐150	60/80/100	53.0	59.0	1107	726	
u111	AF 2	20	90‐120‐150	60/80/100	53.5	114.8	569	410	
u118	AF 1	150	90‐120‐150	60/80/100	53.5	65.9	1002	677	
u119	AF 2	150	90‐120‐150	60/80/100	53.7	93.3	722	451	ss
u214	AF 1	450	90‐120‐105	60‐80‐70	53.9	63.6	993	779	ss
u226	AF 2	450	90‐120‐105	60‐80‐70	53.6	67.7	925	771	ss
u278	AF 5	20	90‐120‐105	60‐80‐70	54.8	104.8	826	465	
u279	AF 5	450	90‐120‐105	60‐80‐70	54.5	60.8	1067	801	ss
u280	AF 5	150	90‐120‐105	60‐80‐70	62.2	122.3	741	461	
u281	AF 5	600	90‐120‐105	60‐80‐70	61.7	46.3	1476	1220	ss
u291	AF 5	300	90‐120‐105	60‐80‐70	61.1	114.7	913	410	ss
u293	AF 6	20	90‐120‐105	90‐80‐70	63.0	127.2	868	414	
u294	AF 5	600	30‐45‐60‐75‐90‐105‐120	120	46.4	50.2	1067	750	*V* = 10 µm/s
u295	AF 6	150	90‐105‐120	60‐70‐80	74.0	161.5	885	299	ss
u297	AF 6	300	90‐105‐120	60‐70‐80	62.8	79.9	1125	632	ss
u300	AF 6	600	90‐105‐120	90‐105‐120	62.4	64.9	1130	823	ss
u301	AF 6	450	90‐105‐120	60‐70‐80	61.1	100.3	729	491	ss
u315	AF5	600	45‐60‐75‐90‐105‐120	120	7.0	6.3	1281	947	*V* = 0.03 µm/s
u319	AF1	600	90‐105‐120	120	52.7	54.4	1027	873	ss
u320	AF2	600	90‐105‐120	120	52.8	79.7	791	653	ss
u321	AF5	600	45‐75‐90‐105‐120‐150	120	4.0	3.4	1298	981	*V* = 0.01 µm/s
u324	AF4	150	90‐105‐120	60‐70‐80	52.8	72.2	1140	610	
u331	AF4	20	90‐105‐120	60‐70‐80	53.0	82.2	1007	550	
u334	AF4	600	90‐105‐120	120	53.5	55.2	1055	867	ss
u335	AF4	450	90‐105‐120	60‐70‐80	53.7	54.1	1046	890	ss
u336	AF4	20	90	60	11.8	31.3	559	300	*V*‐seq2
u339	AF4	300	90‐105‐120	60‐70‐80	58.7	66.7	925	793	ss
u343	AF4	150	90	60	14.1	15.9	1028	817	*V*‐seq2 and ss
u346	AF4	600	90	120	14.2	16.6	894	781	*V*‐seq2 and ss
u349	AF4	300	90	60	14.2	14.9	1004	905	*V*‐seq2 and ss
u350	AF4	450	90	60	14.5	19.2	740	709	*V*‐seq2 and ss

a The velocity‐stepping sequence is 0.3‐1‐3‐10‐30‐100‐300 µm/s or 0.003‐0.01‐0.03‐0.1‐0.3‐1‐3 µm/s, *x* is the load point displacement, *γ* is shear strain (= displacement/instantaneous layer thickness), and *h*_0_ and *h*_f_ are the layer thicknesses at the start and end of shear, respectively.

b Applied normal stress (roughly equal to effective normal stress)

c The ss indicates stick slips occurred at some point during the experiment, *V*‐seq2 indicates the alternative (slow) velocity‐stepping sequence, and *V* = indicates a constant velocity experiment with normal stress stepping.

For the regular experiments, we increased the effective normal stress to either *σ_n_'* = 105 or 120 MPa, with a concomitant increase in pore fluid pressure to *P_p_* = 70 MPa or 80 MPa, initiating stage 2, keeping the pore fluid factor *λ* constant at 0.4 (except for the experiments at 600°C). Again, we sheared the samples at *V* = 10 µm/s, but for only 5 mm of displacement, after which the velocity step sequence was performed. In stage 3, in most experiments, we performed a slide‐hold‐slide (SHS) sequence, with sliding at *V* = 10 µm/s for 1 mm and holds of 3, 10, 30, 100, 300, and 1000 s. The SHS sequence was followed by a velocity step sequence. We will discuss the results of the SHS sequences in a separate contribution. Finally, in stage 4, we increased the effective normal stress to *σ_n_'* = 150 MPa in experiments where this was possible. However, in most cases, shear stress was too high so that shearing at *σ_n_'* = 150 MPa would have overloaded the load cells, so we instead decreased effective normal stress to *σ_n_'* = 105 MPa (and decreased pore fluid pressure to 70 MPa). In later runs (experiment numbers u295 and above), we performed stages 3 and 4 at *σ_n_'* = 120 MPa before stages 2 at *σ_n_'* = 105 MPa (see Table 2). In stage 4, we sheared an additional 5 mm at *V* = 10 µm/s before performing the velocity step sequence. Total displacements varied from *d* ~ 14 mm for the slow experiments with only one velocity step sequence up to *x* ~ 62 mm for the regular experiments with four velocity step sequences, corresponding to shear strains, *γ*, between ~20 and ~100.

## 4 Results

### 4.1 Evolution of Friction Coefficient and Layer Thickness

**Figure 2:**
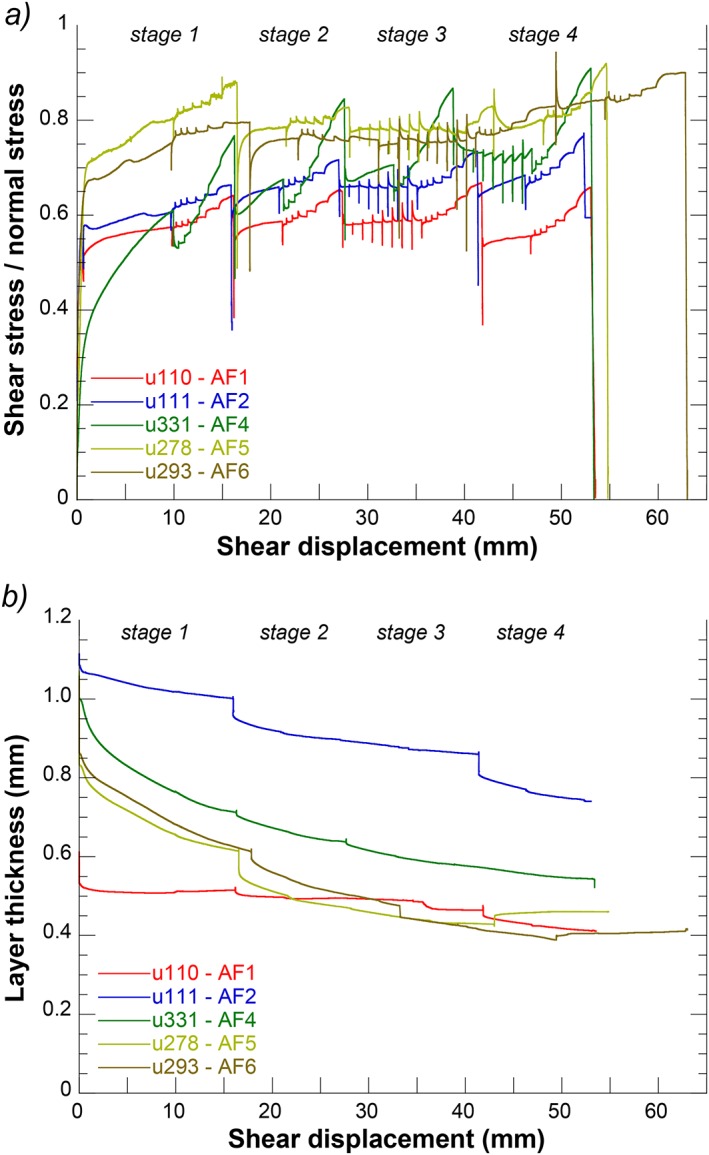
(a) Plot of friction (= shear stress/normal stress) as a function of displacement for regular experiments performed at room temperature. (b) Plot of layer thickness as a function of displacement for the experiments shown in Figure 2a.

We show the friction coefficient (defined as *μ* = *τ*/*σ_n_'*, i.e., shear stress/effective normal stress, thereby ignoring cohesion) as a function of shear displacement for all samples tested at room temperature in Figure 2. All samples show a similar quasi‐linear increase in shear stress with initial displacement, followed by an apparent yield (ultramylonites AF 5 and AF 6) or peak strength (cataclasites AF 1 and AF 2) at displacements of *x* ~ 1–2 mm, with the exception of sample AF 4 (the PSZ 1 from borehole DFDP‐1B, which showed a continuous steady increase in friction over the entire 10 mm of run‐in displacement). Whereas major strain hardening or weakening is absent for all other samples in the remaining stages of the experiments, sample AF4 continued to display strain hardening during all stages, velocity step and the slide‐hold‐slide sequences. All experiments show ongoing layer thinning, due to shear‐enhanced compaction as well as extrusion of material, the latter probably becoming more important at higher displacement (beyond *x* > 2 mm or so, Figure 2b). The layer thinning slows down with increasing displacement; in some cases a steady state layer thickness is achieved which shows dilation upon velocity upsteps (samples AF1, AF5, and AF6).

**Figure 3:**
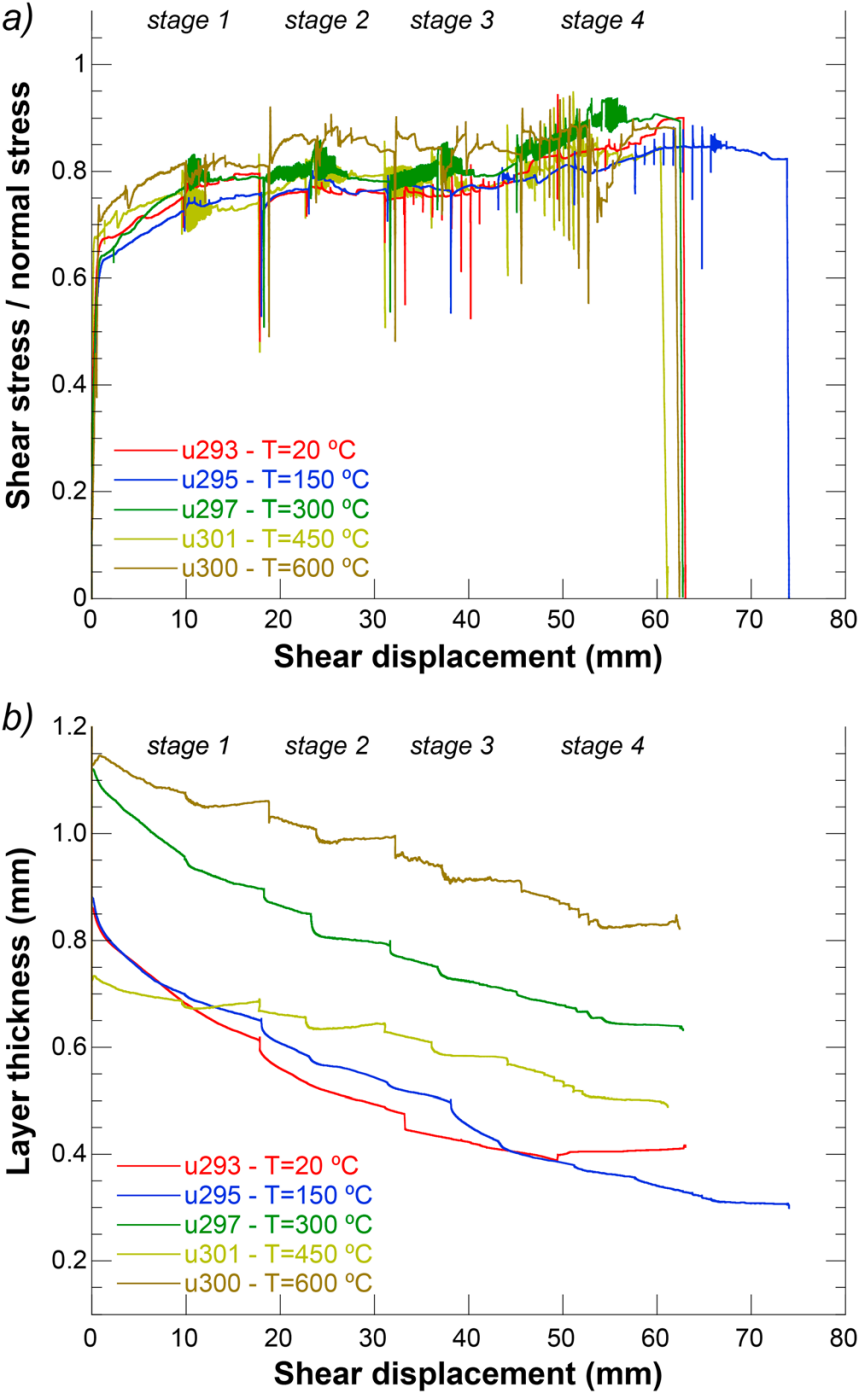
(a) Plot of friction (= shear stress/normal stress) as a function of displacement for regular experiments using sample AF6 performed at temperatures of 20, 150, 300, 450, and 600°C. (b) Plot of layer thickness as a function of displacement for the experiments shown in Figure 3a.

**Figure 4:**
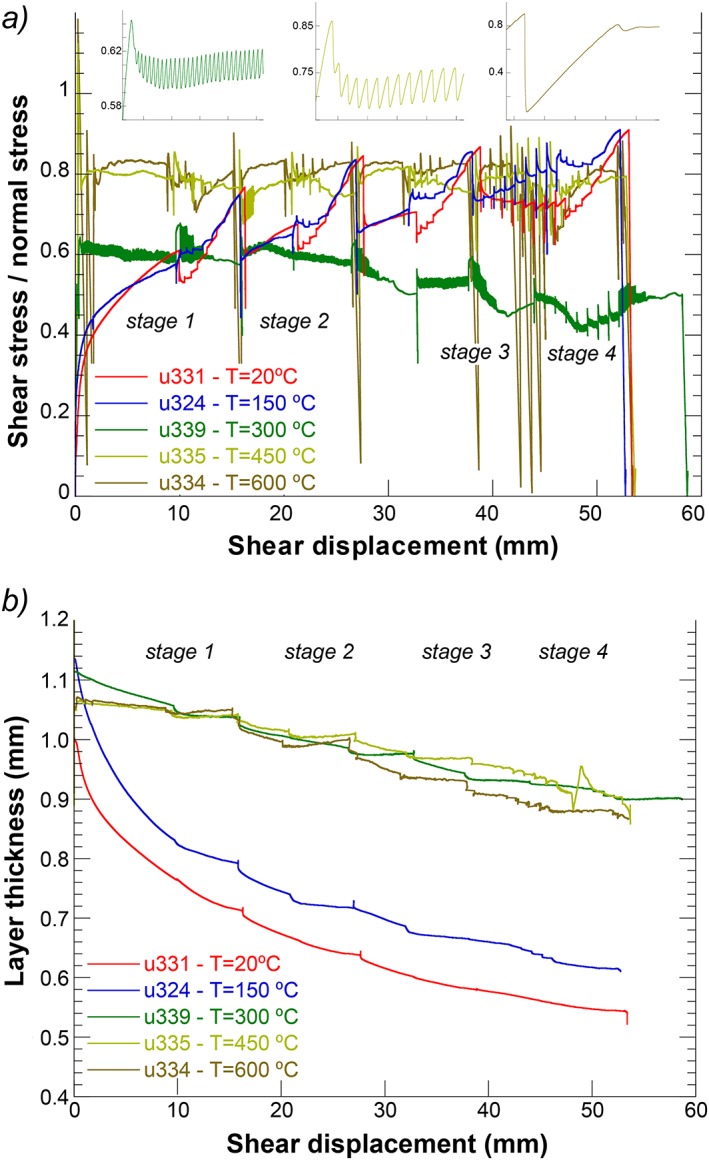
(a) Plot of friction (= shear stress/normal stress) as a function of displacement for regular experiments using sample AF4 performed at temperatures of 20, 150, 300, 450, and 600°C. The insets show stick slips occurring during the run‐in at *V* = 10 µm/s and 105 MPa effective normal stress, at 300, 450, and 600°C, from left to right. (b) Plot of layer thickness as a function of displacement for the experiments shown in Figure 4a.

Figure 3 shows the evolution of friction and layer thickness with displacement for all regular experiments using one of the ultramylonite samples, sample AF6. The results for the sample from the PSZ (AF4) are shown in Figure 4. All samples except for the PSZ‐derived AF4 sample show a similar evolution of friction with displacement at all temperatures tested. Unstable sliding is observed in all experiments at *T =* 300°C and *T =* 450°C, with regular stick slips occurring at velocities from *V =* 1 to 30 µm/s. At *T =* 450°C, stick slips also occur at *V =* 100 µm/s but not consistently. Experiments performed at *T =* 600°C show occasional unstable events (i.e., near‐instantaneous stress drops), but regular stick slips do not occur. The layer thickness shows an exponential decrease with displacement, with no clear variation with temperature.

The results of the experiments on the PSZ (AF4; Figure 4) can be divided in two sets: a low‐temperature set (*T* ≤ 150°C) where friction continually increases with ongoing displacement and a high‐temperature set where friction attains a high peak value (*μ*_peak_ = 1.18 at *T =* 600°C) after a quasi‐linear elastic increase and further displacement hardening does not occur. The difference in the two sets is more clear in the layer thickness evolution (Figure 4b). The layer thickness reduction (*∆h*) is much larger in the two low‐temperature experiments (*∆h* ~500 µm at *T* ≤ 150°C versus ~150 µm at *T* > 150°C) and follows an exponential decay with displacement. Sliding is unstable in the experiment at *T =* 300°C showing regular stick slips throughout the experiment at all load point velocities except for *V* = 0.3, 100, and 300 µm/s (Figure 4a). In addition, the lowest average friction coefficient for AF4 is observed in the *T =* 300°C experiment, with the exception of the initial stages of the low‐temperature experiments. Stick slips occur at *T =* 450°C but they disappear after two or three cycles. Extremely large stress drops occur at *T =* 600°C during the run‐in sections of each phase and the SHS sequence. The maximum shear stress drop observed is *∆τ* ~ 105 MPa in u334 concomitant with a layer thickness decrease of *∆h* ~ 5 µm.

**Figure 5:**
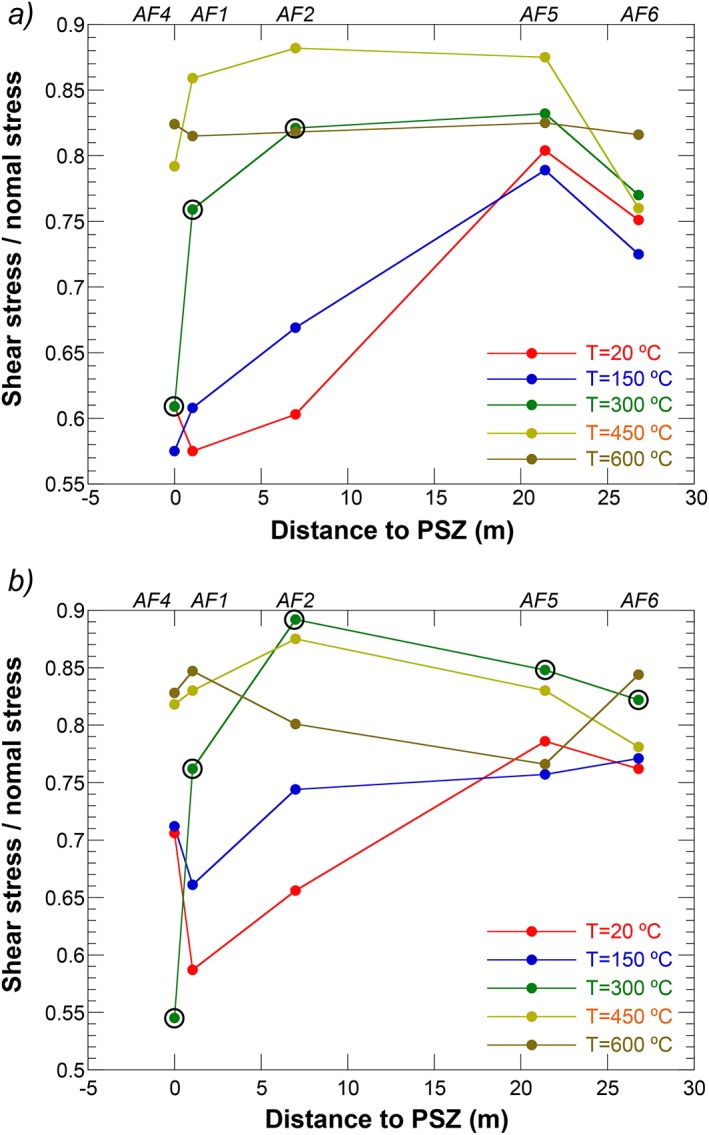
(a) Friction of Alpine Fault samples as a function of distance to the PSZ (see Table 1) for an effective normal stress of 90 MPa and temperatures of 20, 150, 300, 450, and 600°C. Friction was determined at the end of the 10 mm run‐in at *V* = 10 µm/s. Data points obtained from the peak friction during unstable sliding (stick slips) are circled. (b) Friction of Alpine Fault samples as a function of distance to the PSZ (see Table 1) for an effective normal stress of 120 MPa and temperatures of 20, 150, 300, 450, and 600°C. Friction was determined at the end of the 5 mm run‐in at *V* = 10 µm/s. Data points obtained from the peak friction during unstable sliding (stick slips) are circled.

Friction coefficients show considerable variation with temperature for all samples (Figure 5). The largest variation in friction coefficient with temperature is apparent for the samples derived from the cataclasites (AF1 and AF2) and the sample derived from the PSZ (AF4). Friction coefficients at 90 MPa range from *μ* ~ 0.57 at *T =* 20°C (AF1) and *T =* 150°C (AF4) to *μ* = 0.89 at *T* = 450°C (AF2). Samples derived from ultramylonites display a smaller variation between *μ* ~ 0.72 at *T* = 150°C (AF6) and *μ* ~ 0.88 at *T* = 450°C (AF5) and *σ_n_' =* 90 MPa. In general, friction roughly increases with increasing temperature, but samples are typically strongest at *T* = 450°C. Increasing the effective normal stress to *σ_n_' =* 120 MPa changes the picture slightly; we see identical variability in friction with temperature, but now the highest friction is at 600°C or 300°C (Figure 5b). All samples deformed at *T* = 300°C show higher friction at *σ_n_' =* 120 MPa than at *σ_n_' =* 90 MPa. One should bear in mind that these values were derived from the peak values in stick‐slip cycles, which are probably not directly comparable to the values derived from steady state sliding.

AF4, derived from the PSZ, shows considerably higher friction values at *σ_n_' =* 120 MPa than *σ_n_' =* 90 MPa at temperatures of *T* = 20 and 150°C, presumably due to displacement hardening (Figure 4a). At *T* = 300°C, this sample is weaker at *σ_n_' =* 120 MPa than at *σ_n_' =* 90 MPa (note again that the sample was stick slipping through out and that the peak friction values are shown in Figure 5).

### 4.2 Velocity Dependence of Friction (*a‐b*)

**Figure 6:**
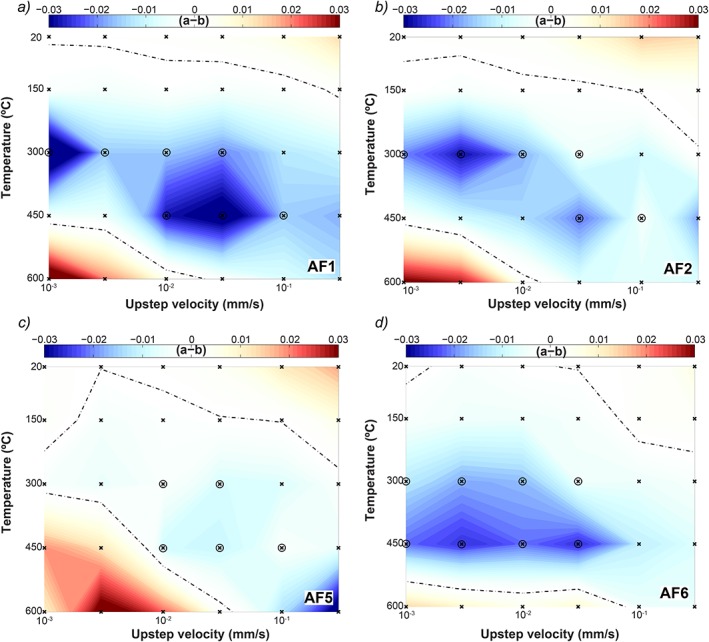
Plot of the velocity dependence of friction, (*a‐b*), as a function of poststep sliding velocity and temperature at 90 MPa effective normal stress for samples (a) AF1, (b) AF2, (c) AF5, and (d) AF6. Data has been interpolated and contoured using the same color scale for all plots. The dashed lines indicate a zero value for (*a‐b*). Data obtained from velocity steps showing stick slips are circled.

**Figure 7:**
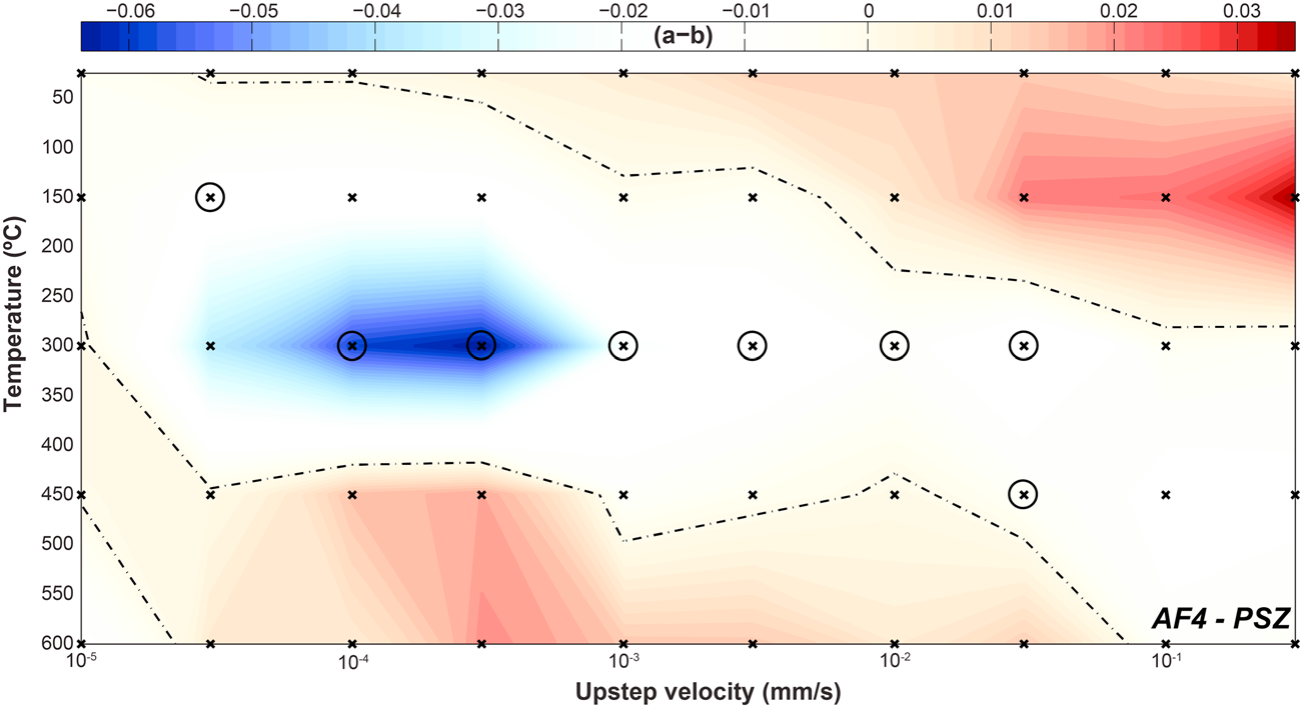
Plot of the velocity‐dependence of friction, (*a‐b*), as a function of poststep sliding velocity and temperature at 90 MPa effective normal stress for sample AF4. Data have been interpolated and contoured. Note that the color scale is different than those in Figure 6. The dashed lines indicate a zero value for (*a‐b*). Data obtained from velocity steps showing stick slips are circled.

We show the values of (*a‐b*) as obtained from model inversions to the data in Figures 6 and 7. Our model inversions include steps that resulted in unstable stick slips. Stick‐slip instabilities impair RSF modeling and often yield models that have no solution and stop the inversion. This is particularly the case for situations where the velocity step occurs directly after or right before a slip event. In those cases, it was necessary to move the modeled point of the velocity step forward or backward to obtain a solution. Although stick‐slip instabilities only occur in velocity‐weakening materials, the magnitude of the (*a‐b*) values obtained from these steps should be treated with caution. We present the data as contour plots, interpolating between points; this allows for a quick inspection of trends with sliding velocity and temperature (Figures 6 and 7).

Both velocity and temperature affect the values of (*a‐b*) (Figure 6). At room temperature, all samples show mostly positive values within the range −0.0029 to +0.0182, with the values increasing with the magnitude of the upstep sliding velocity. This trend is still visible at *T* = 150°C, but for a narrower range of (*a‐b*) values, −0.0048 to +0.007, and with more negative values at upstep velocities of *V* ≤ 10 µm/s. At *T* = 300°C, we observe only negative values for (*a‐b*), again with higher values (i.e., less negative) at higher velocities. In contrast, at *T* = 450°C the largest values for (*a‐b*) are observed at the lowest upstep velocities, being positive for sample AF5 and only slightly negative for samples AF1 and AF2. Sample AF6 is an exception to this trend, as values for (*a‐b*) are similar for all velocities *V* < 100 µm/s. Finally, at *T* = 600°C, all samples show positive (*a‐b*) values at velocities below *V =* 10–30 µm/s, with values up to 0.0361, and negative values at higher sliding velocity. At *T* = 600°C, higher sliding velocities promote velocity‐weakening behavior.

In light of the strong dependence of (*a‐b*) on sliding velocity, we performed a number of longer duration (slow) velocity‐stepping experiments on the PSZ‐derived sample, in which we stepped the sliding velocity from *V =* 3 nm/s to 3 µm/s in half order of magnitude increments at *σ_n_'* = 90 MPa (Table 1). Note that an additional 1:100 gear box was added to be able to employ these low velocities. The results for the (*a‐b*) values are shown in Figure 7, combined with the data from the regular experiments at *σ_n_'* = 90 MPa. Note that the scale bar is different from those in Figure 6, due to the larger spread in *(a‐b)* values; in particular, there are strongly negative values at *T =* 300°C and *V =* 0.1 and 0.3 µm/s. In contrast to the slow experiment, sliding at *V =* 0.3 µm/s in the regular experiment was stable. The values of (*a‐b*) overlapping the two experiments (i.e., steps from *V =* 0.3–1 and *V =* 1–3 µm/s) are within ~0.002 of each other for the experiments at *T* = 20 and *T* = 150°C. The difference is larger at *T* = 300°C, namely, ~0.01, but both show negative (*a‐b*) values. The experiments at *T* = 450 and *T* = 600°C show contrasting behavior in the two types of experiments; (*a‐b*) is strongly positive (up to 0.061) in the slow experiment, whereas it is only slightly positive or negative in the regular experiment. We use the values obtained in the regular experiments (Figure 7) to compare the different samples. Overall, the (*a‐b*) values for the AF4, PSZ‐derived sample, show a similar dependence on temperature and upstep sliding velocity as the other samples (Figure 6). The values at *T* = 300°C are negative for all upstep sliding velocities and are typically characterized by regular stick slips. Similar to the other samples, (*a‐b*) in AF4 shows a broad increase with increasing sliding velocity at temperatures below *T* = 300°C, although there is not a clear trend. At a temperature of *T* = 450°C, (*a‐b*) shows a broad maximum around upstep velocities of *V =* 0.1–0.3 µm/s and decreases to negative values at higher velocities. At a temperature of *T* = 600°C, the maximum in (*a‐b*) is shifted to upstep velocities of *V =* 1–3 µm/s, with (*a‐b*) decreasing to negative values for both lower and higher velocities (Figure 7).

### 4.3 Normal Stress Dependence of Shear Stress at 600°C

**Figure 8:**
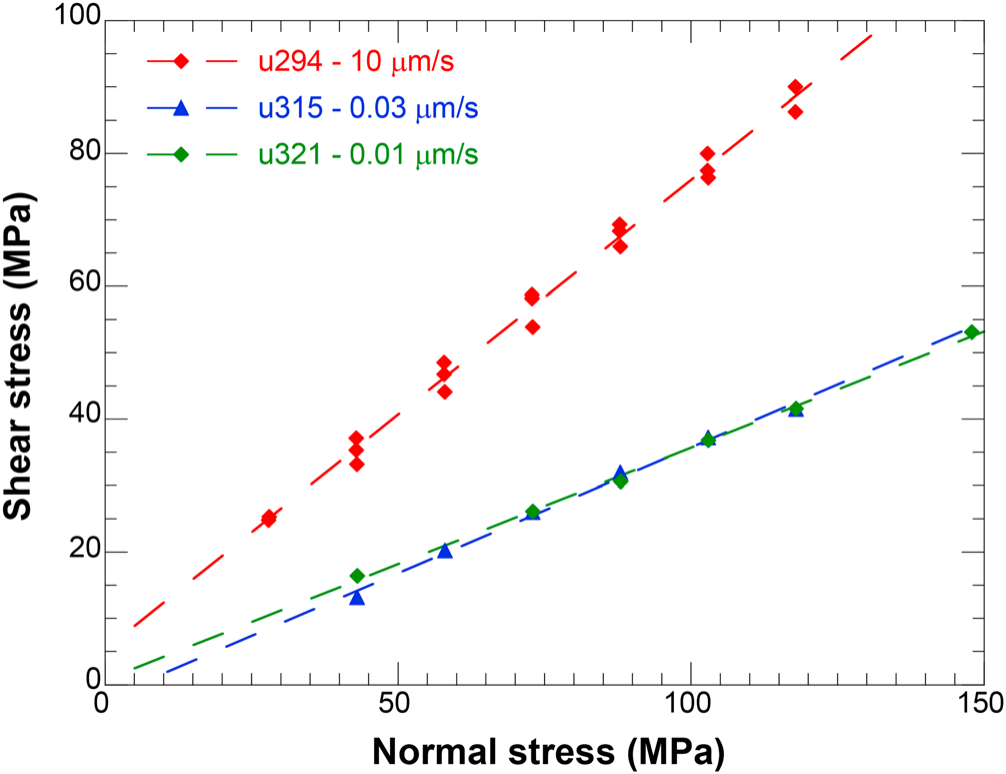
Plot of shear stress as a function of effective normal stress for three experiments performed at 600°C and sliding velocities of *V* = 0.01, 0.03, and 10 µm/s. All data can be fitted with a linear fit; the fitting parameters are listed in Table 3.

To explore the possibility of deformation by plastic processes at high temperature, we performed three experiments at constant sliding velocity, stepping effective normal stress in the range *σ_n_'* = 30–150 MPa, using the ultramylonite, AF5. The results are summarized in Figure 8, which shows the steady state shear stress (*τ*) as a function of effective normal stress (*σ_n_'*) for all three sliding velocities investigated. All data show a linear increase in shear stress with effective normal stress with goodness of fit values (*R* ^2^) better than 0.99. The fit parameters (Table 3) show a physically impossible negative cohesion value for the experiment performed at 0.03 µm/s. However, our correction for the seal friction inevitably leads to an additional error in the shear stress and normal stress, which we estimate to be around 1 MPa. Therefore, the values of the intercepts (i.e., the cohesion) should be treated with some caution.

**Table 3:** Parameters From Linear Regression Fits to Normal Stress Stepping Data

Experiment	*V* (µm/s)	*μ*	SE	Cohesion (MPa)	SE	*R* ^2^
u294	10	0.707	0.015	5.280	1.152	0.993
u315	0.03	0.379	0.014	−2.121	1.204	0.994
u321	0.01	0.350	0.008	0.693	0.832	0.998

### 4.4 Post Deformation Observations

After each experiment, the piston assembly was removed from the vessel and dried overnight at *T* = 50°C. Subsequently, the top piston and outer confining ring were carefully removed. In most cases, the gouge layer split apart on radially emanating fractures upon removal of the outer confining ring, in an orientation similar to Riedel (R1) fractures. Several chips of about 10–15 mm long were recovered, aligned, and set in Araldite 20/20 epoxy resin. For slow experiments (i.e., *V* < 1 µm/s), the confining rings and gouge layer were recovered from the pistons in one piece (supporting information Figure S3a). The gouge layer was pushed out of the confining rings, which caused it to fragment in several pieces, often on angled fractures (supporting information Figure S3b), which were again aligned and set in epoxy.

We would expect to be able to deduce deformation mechanisms from microstructural observations of these samples, but because the sliding and normal stress history of our samples is complex, it is difficult to infer the dominant deformation processes and at which point(s) during the experiment they were operating. Constant velocity experiments, to a predescribed displacement or strain, are required to systematically investigate changes in operating deformation mechanisms but are beyond the scope of the present work.

To highlight some general deformational aspects, we show microstructures recovered from the normal stress‐stepping experiments on sample AF5 at constant velocity (see also Tables 1 and 3). We emphasize that the microstructural work presented here is incomplete but serves to highlight contrasts between samples deformed slow (*V* = 0.03 µm/s) and fast (*V* = 10 µm/s), particularly with respect to the degree of localization and grain size reduction.

**Figure 9:**
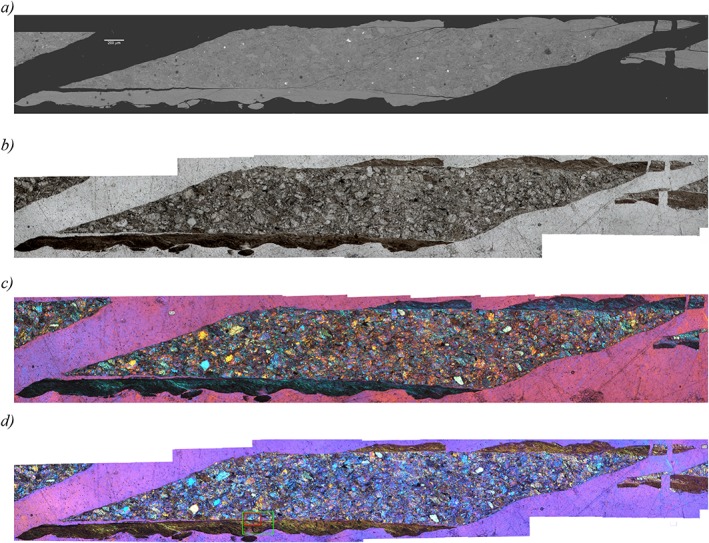
(a) Mosaic of backscatter electron images of sample AF5, experiment u294, sheared at *V* = 10 µm/s, 600°C and variable normal stresses and fluid pressures. Shear sense is top to the left. (b) Same as Figure 9a but with images from the light microscope. (c) Same as Figure 9a but with images from the light microscope with crossed nicols and with the gypsum plate inserted. (d) Same as Figure 9c but with a rotation of 90°. Red box indicates the approximate location of Figures 10a and 10b; the green box indicates the approximate location of Figure 10c.

**Figure 10:**
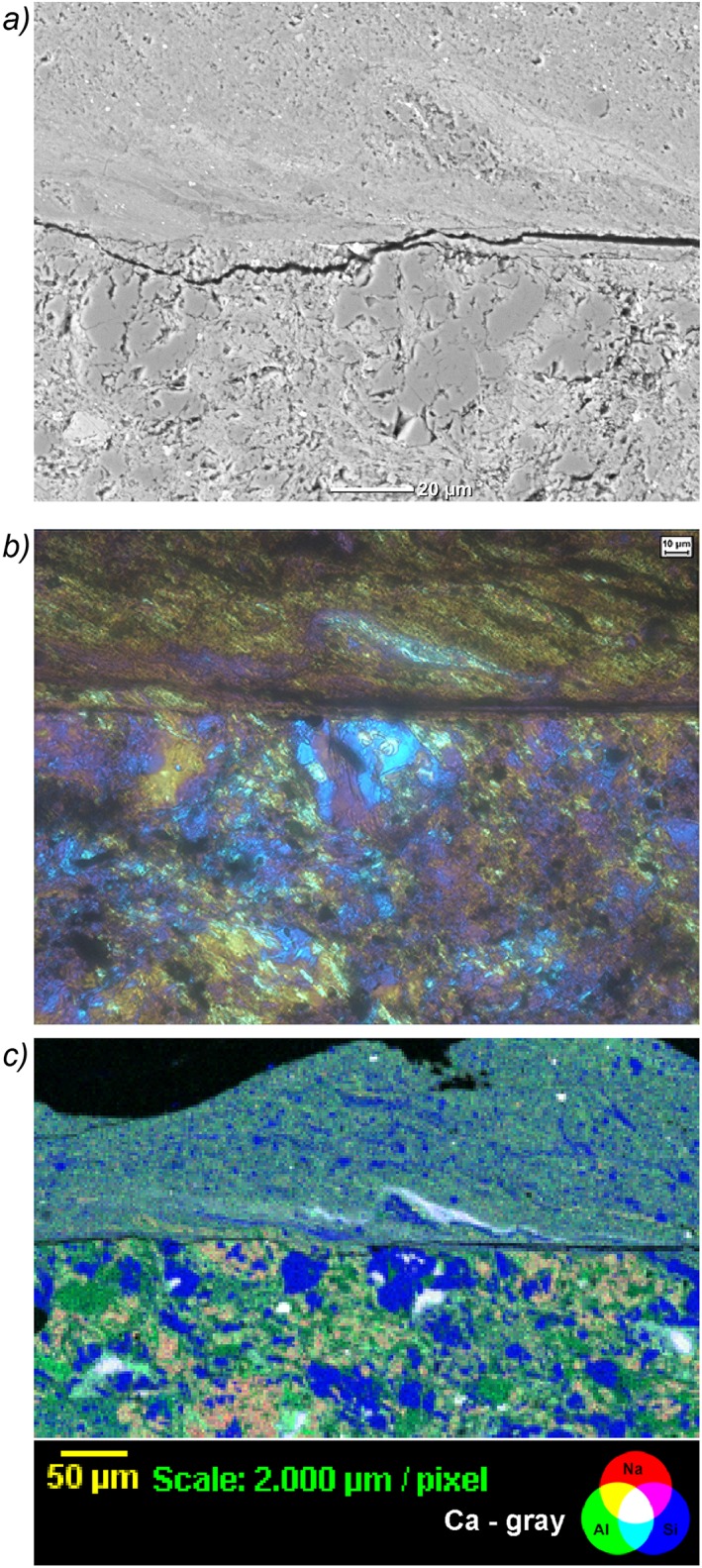
(a) Backscatter electron images of sample AF5, experiment u294, sheared at *V* = 10 µm/s, 600°C and variable normal stresses and fluid pressures. Shear sense is top to the left. *Q* = quartz and *C* = calcite. (b) Light microscope image of the same area as shown in Figure 10a, with crossed nicols and with the gypsum plate inserted. (c) RGB (red‐green‐blue) composite image of element maps of Si, Al, Na, and Ca obtained using wavelength dispersive X‐ray spectroscopy on an electron micropobe.

The microstructure of the gouge deformed at *V =* 10 µm/s shows two boundary‐parallel shear bands (B shears, Figure 10), defined by a much finer average grain size than in the remainder of the gouge (<1 µm versus 5–50 µm). The thickness of the B shear bands varies between ~70 and ~150 µm but is not continuous along the sample length, most likely because it remained stuck to the piston (Figure 9). The boundary with the remainder of the gouge is quite sharp and flat, in contrast to the boundary with the piston interface, which reflects the roughness of the piston (Figures 9 and 10). The larger grains in the matrix of the gouge measure up to 100 µm in diameter and are subangular to rounded in shape with an apparent random orientation. These larger grains are always quartz and are visible as fragmented shapes which form a jigsaw texture (Figure 10a). Calcite grains inside the shear band have sigmoidal shapes and are up to 40 µm long. Feldspar is very fine grained (1 µm and below), both in the shear band and in the matrix of the gouge (Figures 10b and 10c). Phyllosilicates are extremely fine grained and do not align to form an obvious foliation. When viewed in crossed polarized light with the gypsum plate inserted, the boundary shear shows a more or less uniform color (Figures 9c and 9d ). When the stage is rotated 90°, the color changes uniformly. However, when viewed in closer detail (Figure 10b), the boundary shear is not uniform, showing an elongated calcite grain with a sigmoidal shape. The uniform extinction occurs in streaky bands at an angle of about 25–30°; the extremely fine grain of the phases forming the uniform extinction prohibits identification of the responsible mineral (Figure 10c). We have not yet attempted to obtain an EBSD pattern, since it is likely that the grain size is too small to yield reliable results.

**Figure 11:**
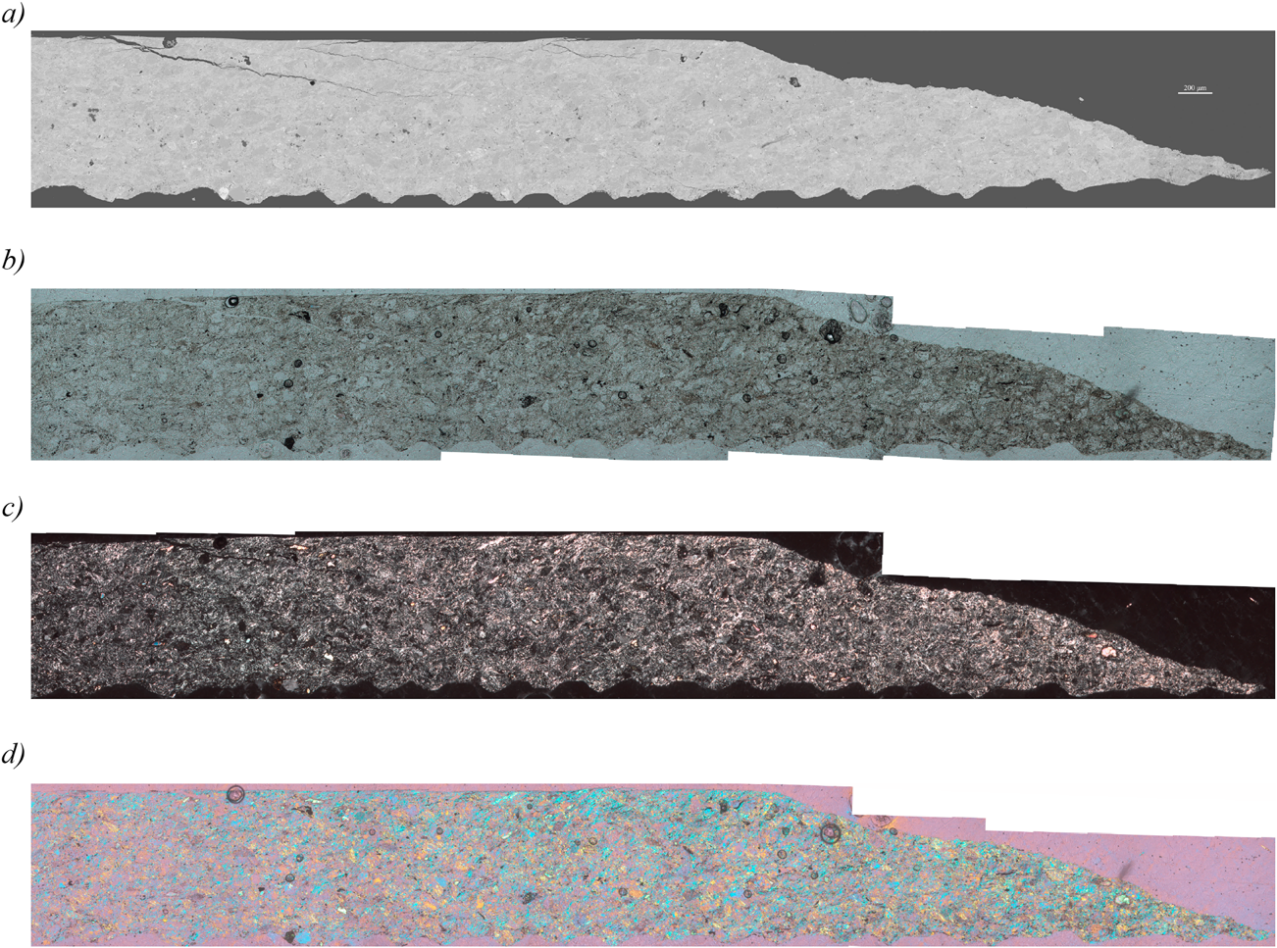
(a) Mosaic of backscatter electron images of sample AF5, experiment u315, sheared at *V* = 0.03 µm/s, 600°C and variable normal stresses and fluid pressures. Shear sense is top to the right. (b) Same as Figure 11a but with images from the light microscope. (c) Same as Figure 11a but with images from the light microscope with crossed nicols. (d) Same as Figure 11a but with images from the light microscope with crossed nicols and with the gypsum plate inserted.

The microstructure of the sample sheared at low velocity of *V* = 0.03 µm/s lacks obvious localization features such as boundary or Riedel (R1) shears (Figure 11). The imprint of the teeth is visible on the lower boundary but absent on the top boundary. This might indicate the presence of a boundary shear here, but there are no obvious changes in grain size or mineralogy with increasing distance from the boundary. The microstructure of the gouge appears quite homogenous with only one larger fracture with an R shear orientation. Larger grains of quartz are easily recognizable both in thin section and in the SEM. These quartz grains measure up to 100 µm in size and show some fractures but are less fragmented than the grains in the fast experiment. There is no obvious preferred grain shape orientation. As in the previously described section, individual feldspar grains are difficult to identify and are much finer grained (<5 µm) than the quartz grains. The phyllosilicate grains are similarly fine grained, but when viewed with crossed nicols or crossed nicols with the gypsum plate inserted (Figures 11c and 11d), a weak foliation with a P shear orientation is recognized [*Logan et al*., 1992].

## 5 Discussion

### 5.1 Frictional Strength of the Alpine Fault

Previous experimental work has already shown that samples derived from the central Alpine Fault zone are frictionally relatively strong, with steady state, stable *μ* = 0.6–0.8 [*Boulton et al*., 2012, 2014; *Ikari et al*., 2014, 2015]. Although comparison with these previous studies is complicated by differences in experimental configuration, temperature, and effective normal stress, it is important to constrain variations in friction between different studies. Our weakest sample is derived from the smectite‐bearing PSZ. Our room temperature friction coefficient for this sample, *μ* = 0.67, is significantly higher than the value of *μ* = 0.43 reported by *Boulton et al*. [2014]. However, our value is determined at higher effective normal stress (*σ_n_'* = 90 MPa versus *σ_n_'* = 31.2 MPa) and we observed significant strain hardening, which might be explained by a preferential displacement‐induced extrusion of fine‐grained smectite (note the much larger decrease in layer thickness in Figure 4). If smectite is preferentially extruded through the miniscule gaps existing between the pistons and the coated confining rings, then this continuously changes the composition of the fault gouge, which somewhat limits the applicability of the results; i.e., the values of friction and (*a‐b*) do not necessarily reflect the starting composition. At the same time, however, the determination of exact clay content is nontrivial and the results for the PSZ at temperatures above 150°C do not show the same typical hardening trends. This last point indicates that the extrusion of smectite either did not occur in the higher temperature experiments or that it did not affect strength. The value of *μ* measured at a small displacement of *d* = 1.5 mm (*γ* ~ 2) is 0.45, which is similar to the value of *μ* = 0.43 obtained by *Boulton et al*. [2014] and to that of *μ* = 0.45 obtained by *Ikari et al*. [2014] at *σ_n_'* = 25 MPa.

At temperatures below *T* = 300°C, the cataclasite‐derived samples are consistently weaker than those derived from mylonites (Figure 5). Mineralogically, semiquantitative X‐ray diffraction does not show obvious differences between these two sample sets. However, phyllosilicate contents are difficult to quantify reliably, particularly for disordered mineral structures, so it is possible that the slightly lower friction coefficient for the cataclasite‐derived samples result from a higher phyllosilicate content. Higher phyllosilicate contents for samples from closer to the PSZ were inferred by *Ikari et al*. [2014] and are in line with petrologic, geochemical, and geophysical observations of fluid‐rock interactions throughout the cataclasites [*Sutherland et al*., 2012; *Townend et al*., 2013; *Menzies et al*., 2014; *Toy et al*., 2015].

All samples deformed at *T* = 300°C showed unstable stick‐slip behavior, in some cases occurring during the run‐in, and in all cases occurring during the velocity‐stepping sequence at velocities between *V =* 3 and *V =* 30 µm/s. Compared to previous studies, our results of changes in friction coefficient with temperature of the PSZ‐derived sample agree well with those obtained by *Boulton et al*. [2014] and *Ikari et al*. [2014]. A friction coefficient of 0.67 was obtained by *Ikari et al*. [2015] from a sample close to the PSZ of borehole DFDP‐1A, at 160°C and 100 MPa effective normal stress, which is in reasonable agreement with the value obtained here (0.57 at 90 MPa and 0.72 at 120 MPa and 150°C, see Figure 5). In contrast, *Ikari et al*. [2015] observed weakening with increasing temperature in ultramylonite‐derived samples (which are similar to our samples AF5 and AF6), notably when deformed wet. Their dry samples showed consistent strengthening with increasing temperature up to *T* = 230°C, with the exception of one data point at a higher normal stress, which was well below the trend by about 0.2 friction units. Our results do not show a significant dependence of friction on temperature for the ultramylonite‐derived samples (AF5 and AF6). The difference in results might be related to the sample configuration used. *Ikari et al*. [2015] employed a single direct shear method sliding two cylinders over each other, creating shear on a plane rather than shear within a volume as in our experiments.

### 5.2 Velocity Dependence of Friction

As in previous studies [*Boulton et al*., 2014; *Ikari et al*., 2014, 2015], the velocity dependence of friction, expressed as (*a‐b*), at room temperature is mildly positive with values ranging from −0.0037 to +0.0182 and with no systematic dependence on sliding velocity, although lower values tend to occur for slower velocity steps. There is no clear difference in (*a‐b*) between the different samples; larger variability occurs as a function of sliding velocity. The variability as well as the absolute magnitude of (*a‐b*) are similar to those observed by *Ikari et al*. [2014] for a much larger sample suite and at 30 MPa effective normal stress, although these authors did not find any negative values.

Elevated temperature increased the range of (*a‐b*) values for all samples investigated, leading to large positive as well as large negative values (Figures 6 and 7). The total range in (*a‐b*) values spans −0.0590 to +0.061; if we exclude the slow steps for the PSZ samples, this range narrows somewhat to −0.0395 to +0.0361. Note here that the strongly negative values are derived from steps that showed stick slips, which are difficult to model (see supporting information Figure S5 for examples of model fits). Sample AF6, a brown‐green‐black ultramylonite, displayed the most negative vales of (*a‐b*) (Unit 2 [*Toy et al*., 2015]). This sample is the furthest away from a principal slip zone and is expected to have the least amount of secondary clays and calcite (Table 1).

Given the overall similarity in sample mineralogy, our results show that temperature and sliding velocity have a larger effect on the value of (*a‐b*) than the minor compositional variations of our samples. All samples show a transition to velocity‐weakening behavior with increasing temperature. The temperature at which this transition occurs depends on the slip velocity: at low slip velocity (*V* < 30 µm/s), the transition occurs at *T* ≤ 150°C, whereas at high slip velocity (*V* ≥ 30 µm/s), the transition occurs at *T* ≥ 300°C. In addition, a second transition back to velocity‐strengthening behavior is observed at *T* > 450°C or *T* = 600°C (depending on the sample) but only at lower sliding velocities (*V* < 100 µm/s). No clear systematic relationship between (*a‐b*) and sliding velocity or temperature is apparent.

A transition to velocity weakening with increasing temperature has been reported for many fault rocks and analogue mixtures [e.g., *Chester and Higgs*, 1992; *Chester*, 1994; *Blanpied et al*., 1995, 1998; *He et al*., 2007, 2013; *Niemeijer et al*., 2008; *den Hartog et al*., 2012b, 2013; *Niemeijer and Collettini*, 2013; *Niemeijer and Vissers*, 2014; *Verberne et al*., 2013; *Boulton et al*., 2014; *Pluymakers et al*., 2014]. In several studies, the transition temperature was also found to depend on the sliding velocity. All of these studies were conducted under hydrothermal conditions in the presence of a reactive fluid, which suggests that the transition to velocity weakening is related to the thermal activation of a fluid‐assisted deformation mechanism. Our general observations fit with predictions of a phenomological model wherein a competition between dilatant, slip‐dependent granular flow and a thermally activated, time‐dependent compactive creep mechanism controls velocity dependence [*Niemeijer and Spiers*, 2006, 2007, N‐S model hereafter]. It is likely that a similar competition controls porosity, and hence the onset of velocity weakening, in Alpine Fault rocks as well.

However, our new results suggest that the details of the processes are more complex than the simple two‐phase model of distributed deformation described by *Niemeijer and Spiers* [2007]. The addition of extra phases, such as feldspars that are prone to cataclastic failure along cleavage planes, as well as the presence of a high strength, rough boundary, affect the microstructural evolution (Figures 10 and 11) and, presumably, friction and its velocity dependence. In particular, localized zones with extreme grain size reduction such as those in Figure 9 are expected to control frictional strength [e.g., *Logan et al*., 1992]. We postulate that the localized grain size reduction along the boundary with the rough simulated “wall rock” is due to higher‐than‐average contact stresses at these contact points with the hard Ni alloy. At low sliding velocities, the gouge material can relax the shear and normal stresses by growth of the contact area through a time‐dependent creep mechanism, but this process either is not possible or occurs too slowly to operate at higher sliding velocity.

In the N‐S model, a finer grain size will lead to more time‐dependent compaction, lower porosity, and thus higher strength. At the same time, a finer grain size results in larger areas of contact between the different mineral phases, including the potentially weak phyllosilicates [*Niemeijer and Spiers*, 2006; *Moore and Lockner*, 2011; *Giorgetti et al*., 2015]. This in turn could allow more slip to occur on weaker phyllosilicates, with the intervening material able to accommodate slip through a time‐dependent mechanism, such as pressure solution, effectively leading to frictional weakening similar to the microphysical model of *Bos and Spiers* [2002, B‐S model hereafter]. In samples with localized grain size reduction (Figure 9), very fine grain size in the boundary shear zone should allow for very rapid grain boundary diffusion, but even solid state diffusion might occur quickly in nanograins [*Verberne et al*., 2014].

The combination of frictional granular flow with a viscous time‐dependent slip accommodation mechanism can result in a strong velocity dependence of frictional strength as well as a strong velocity dependence of (*a‐b*). Overall, the transition from velocity strengthening to velocity weakening depends on the kinetics of the viscous, time‐dependent deformation accommodating mechanism. Thus, the velocity dependence of friction and the individual values of the RSF parameters (*a*, *b*, and *d_c_*) depends on both temperature and sliding velocity, as well as sliding history through the development of a foliation, possible grain size reduction, and environmental factors such as lithology, effective normal stress, and pore fluid chemistry [*Bos and Spiers*, 2002; *Niemeijer and Spiers*, 2007].

### 5.3 Unstable Slip in Velocity‐Strengthening Material at 600°C

All experiments performed at a temperature of *T* = 600°C show sudden stress drops during the run‐in, i.e., when sliding at *V =* 10 µm/s. The magnitude of these stress drops varies from sample to sample and is largest for the PSZ‐derived sample. At the same time, the velocity step of *V =* 3–10 µm/s in all cases shows mildly to strongly velocity‐strengthening behavior (0.002 < (*a‐b*) < 0.0219, Figures 6 and 7). The unstable slip events occur without any clear correlation to the amount of displacement accommodated and are isolated events, happening at most 4 times during the *d* = 10 mm run‐in. They also occur during the run‐ins of the other stages, with no systematic change in stress drop. The stress drop observed during the *V =* 3 µm/s run‐in in the slow experiments is ∆*τ* ~ 24 MPa, about 25% of the stress drop at *V =* 10 µm/s. This is in contrast to regular stick‐slip events, the stress drop of which increases with decreasing sliding velocity, presumably due to more healing during the arrest phase at low driving velocity [*Ohnaka*, 1973; *Shimamoto and Logan*, 1986; *Wong and Zhao*, 1990; *Karner and Marone*, 2000]. It is unlikely that the smaller stress drop observed at *V =* 3 µm/s is related to a change in stiffness of the apparatus due to the addition of an extra gear box, since the slopes of the quasi‐linear load‐up leading to failure are similar for the two experiments.

These observations point to an origin of the unstable slip events at *T* = 600°C (and possibly some at *T* = 450°C) that is different from a “classical” rate‐and‐state frictional instability and suggests the involvement of pore fluid pressure. All samples contain hydrous (phyllosilicate) minerals which may release water during heating. The onset of the transformation of smectite to illite occurs in natural settings at temperatures as low as 58°C and is completed at temperatures up to 142°C [*Freed and Peacor*, 1989]; the temperatures depend on several factors, such as pore fluid chemistry, smectite crystallinity, and porosity. However, this mineral transformation is not instantaneous; thus, the short duration of our experiments makes it probable that smectite continues to exist at *T* = 150°C, but it is unlikely to still be present as a pure phase at temperatures above that [*Schleicher et al*., 2015]. In the absence of fluids, illite starts to dehydrate at *T* = 150°C and to dehydroxylate at *T* = 550°C [*Gualtieri and Ferrari*, 2006]. Muscovite dehydroxylation occurs at a temperature of *T* = 600°C but is suppressed by the presence of high pore fluid pressure [*Mariani et al*., 2006]. Calcite can react with quartz to form wollastonite plus CO_2_ at a temperature of *T* = 600°C, provided that the CO_2_ pressure is low [*Harker and Tuttle*, 1956].

**Figure 12:**
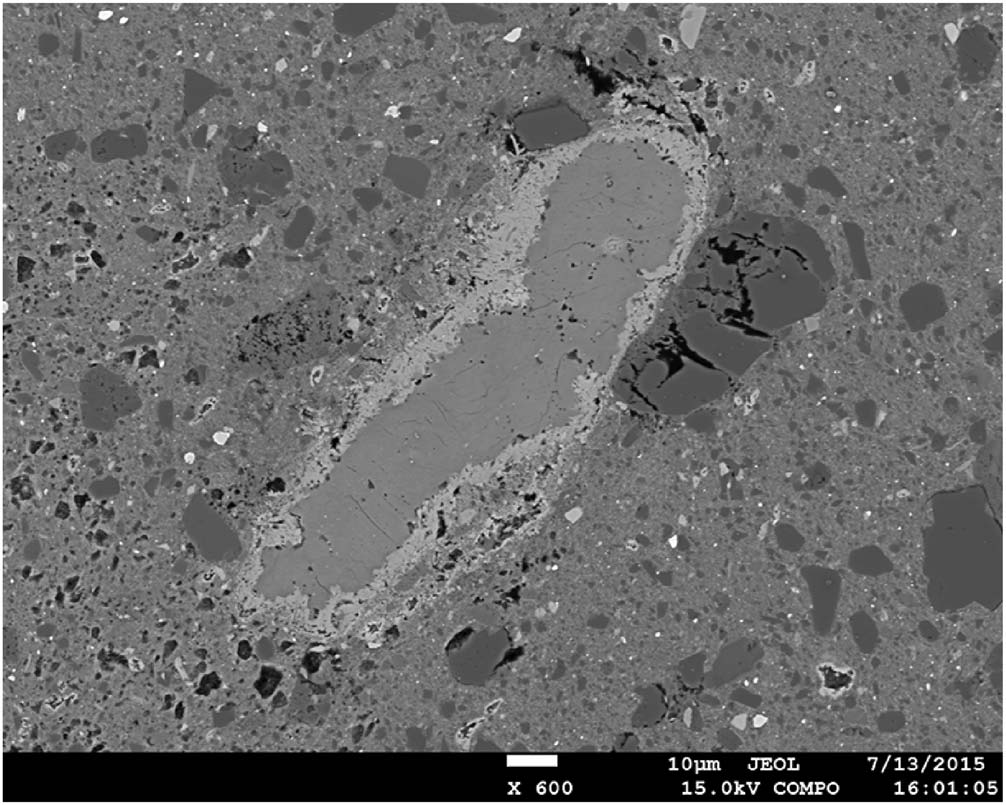
Microprobe image of sample u346, sheared at 600°C, an effective normal stress of 90 MPa, a fluid pressure of 120 MPa, and variable sliding velocity from *V* = 0.003 to 3 µm/s, showing a calcite grain with a reaction rim bearing Ca and Si, presumably wollastonite.

It is possible that one or more of these reactions took place and released a fluid phase, but the question is whether reaction kinetics allow for them to occur during the 1–2 h of equilibration before the start of shear. Products of dehydroxylation reactions are not easy to detect, but we did observe reaction rims consisting of Ca and Si around large calcite grains in the PSZ‐derived sample deformed at *T =* 600°C for ~76 h (Figure 12), which were not present in the starting material. In addition to fast kinetics, a low permeability of the fault gouge is required to allow for local pore pressure buildup, since our experiments are drained. We can calculate a characteristic diffusion time for a pore pressure pulse to decay using

(4) $$t=\frac{h^{2}\beta_{p}\eta }{2k}$$

where *h* is the diffusion distance, *β_p_* is the compressibility of the porous matrix, *η* is the viscosity of the fluid, and *k* is the permeability [*Ikari et al*., 2009]. The shortest distance for the diffusion of pore fluid in our experimental setup is perpendicular to the experimental fault, but this is typically the least permeable direction for faults [e.g., *Wibberley and Shimamoto*, 2003]. We calculated the diffusion time for a range of parameters and for diffusion distances equivalent to flow along and perpendicular to the fault (Figure 14). This figure shows that for low enough permeabilities (e.g., *k* < 10^−19^–10^−20^ m^2^, values determined experimentally for gouges from the Alpine Fault [ *Boulton et al*., 2012; *Carpenter et al*., 2014] and comparable to the value inferred from fluid pressure measurements in the DFDP‐1 borehole [*Sutherland et al*., 2012]), pore fluid pressure should be able to increase in the duration of the experiment. Despite the potential for pore fluid overpressures at 600°C, we measured peak shear stresses that correspond to friction values above 1 for the applied effective normal stress. This means that if high pore fluid pressure developed, it must have been highly localized without influencing the overall effective normal stress. Failure of these high pore fluid pressure patches would give an almost instantaneous drop in effective normal stress, leading to fast, unstable slip.

From the sudden acceleration and deceleration of slip, we observe that the duration of the slip event is similar for both driving velocities (0.8 ms, measured at 5 kHZ recording rate). If slip duration is perhaps limited by the diffusion time of high pore pressures, the stress drop in the case of the low driving velocity is expected to be lower. However, the precise mechanism of nucleation of the unstable slip event remains unproven and more work is needed. In addition, smectite dehydration is probably not relevant for the Alpine Fault, since the fault rocks are being exhumed and are thus likely to experience the reverse, retrograde reactions. However, depressurization during exhumation has been suggested to lead to dehydroxylation of chlorite and, where present, epidote phases, which could potentially have the same effect [*Vry et al*., 2009]. The rapid exhumation rates observed along the central Alpine Fault leads to an almost isothermal drop in pressure which could drive devolatilization reactions of chlorite and epidote. Assuming that permeability of the mylonitic rocks under these conditions is quite low (*k* < 10^−20^ m^2^, e.g., *Wibberley and Shimamoto* [2003]), high pore fluid pressures could develop locally. In turn, these could cause acceleration of slip as the effective normal stress is decreased. The spatial and temporal distribution of high‐ and low‐pressure zones depends on the thermal structure as well as the mineralogy of the fault zone at depth. In turn, this distribution may control the nucleation and propagation of accelerating and decelerating slip.

Seismic and magnetotelluric anomalies present at depth adjacent to the Alpine Fault have been correlated with the presence of (saline) fluids in permeable networks produced primarily through metamorphic dehydration reactions [*Wannamaker et al*., 2002; *Stern et al*., 2007]. The depth extent of the anomalies, 10–35 km, coincides with the calculated PT conditions of fluid‐releasing reactions of chlorite and epidote [*Vry et al*., 2009]. This inferred depth range overlaps the hypocentral depth of low frequency earthquakes, tremors, and slow slip events (20 km depth) [*Wech et al*., 2012; *Chamberlain et al*., 2014]. These events are hypothesized to involve slow shear slip and fluid pressure‐controlled reductions in effective stress [*Wech et al*., 2012; *Chamberlain et al*., 2014]. The potentially fluid‐induced slip events that we observed in experiments at *T* = 450 and *T* = 600°C, in particular in experiments on the PSZ‐derived material, may be analogous to such seismologically recorded slow slip phenomena (Figure 4 and supporting information Figure S3), since they occur under conditions where the material is velocity strengthening. Maximum slip velocity and/or rupture extent could in turn be limited by the velocity‐strengthening behavior of the deforming material. Scaling our experimental results to natural conditions requires better constraints on the identity and kinetics of the fluid‐releasing reactions and/or detailed experiments on analogous fault rocks under conditions representative of the lower crust (cf. *Lamb et al*. [2015] for a discussion of lithologies comprising the crustal root beneath the Southern Alps).

### 5.4 Seismic‐Aseismic or Brittle‐Plastic Transition

Quantifying the areal extent of the brittle, seismogenic, portion of the Alpine Fault and the likely slip rate behavior of other sections will better constrain estimates of maximum moment magnitude and strong ground motion potential. An unknown variable remains the actual geothermal gradient, and its variations with depth, on the Alpine Fault [e.g., *Craw*, 1997; *Toy et al*., 2010; *Sutherland et al*., 2012]. Our data suggest that the Alpine Fault rocks have the largest potential for unstable slip at a temperature of *T =* 300°C. Even though our results do not preclude the nucleation of unstable, seismic slip at *T* ≥ 300°C, they do suggest that the possibility of a large rupture is much smaller at elevated temperature, due to the strong velocity‐strengthening nature of the material at low sliding velocities/strain rates. The maximum depth of (micro)seismicity of ~8–10 km [*Boese et al*., 2012; *Leitner et al*., 2001; *Bourguignon et al*., 2015] implies an average geothermal gradient of 35–40°C/km, which is compatible with geological estimates using fluid inclusions [*Toy et al*., 2010] but is lower than that inferred from direct measurement in the DFDP‐1B hole, (63°C/km [*Sutherland et al*., 2012]). Such an elevated geothermal gradient over 8–10 km depth would raise the maximum depth of seismicity to less than ~ 5 km, which is not observed. These discrepant observations can be resolved if the geothermal gradient decreases with increasing depth, averaging out to 35–40°C/km over the 8–10 km of the seismogenic zone. In addition, topography may also influence shallow (<1–2 km) variations in geothermal gradient [e.g., *Koons*, 1987; *Craw*, 1997; *Toy et al*., 2010].

Variations in temperature on the Alpine Fault influence both the base level of friction and the velocity dependence of friction (Figures 5, 6, 7). *Chester and Higgs* [1992] and *Chester* [1994] loosely defined three regimes and used an empirical set of rate and temperature‐dependent equations to describe their data, assuming that each regime is dominated by a single deformation mechanism and thus by a single set of constitutive parameters:

(5) $$\mu_{ss}=\mu_{0}+\left(a-b\right)\ln\left(\frac{v}{v_{0}}\right)+\left(\frac{aQ_{a}-bQ_{b}}{R}\right)\left(\frac{1}{T}-\frac{1}{T_{0}}\right)$$

In addition to the regular rate‐and‐state parameters, here activation energies are defined to describe the temperature dependence of the controlling mechanism of the direct (*Q_a_*) and evolution effect (*Q_b_*). To isolate these parameters, temperature‐stepping tests are required. In absence of such tests, *Blanpied et al*. [1995] assumed that they are the same (*Q_a_ = Q_b_ = Q*) and subsequently used this formulation to describe their data for wet Westerly granite, sheared at an effective normal stress of 400 MPa and velocities of 0.01, 0.1, and 1 µm/s (Figure 14a). Three regimes were identified and fit with constant values for (*a‐b*) and *Q* within each regime. A low‐temperature regime 1 (*T* < 100°C) was defined by velocity strengthening and temperature weakening, an intermediate temperature regime 2 (*T* = 100–300°C) by velocity weakening and temperature strengthening, and a high‐temperature regime 3 (*T* > 300°C) by velocity strengthening and temperature weakening.

Our data reproduce one result reported from the hydrothermal Westerly granite and quartz experiments: the strong temperature weakening accompanied with large (*a‐b*) values observed at low sliding velocity and temperatures higher than *T* = 300°C [*Blanpied et al*., 1998, Figure 14]. Note that the weakening only occurs when sliding velocity is lower than *V =* 0.3 µm/s and that this weakening occurs over a relatively large slip displacement. Linear fits of friction with 1/*T* give values of 400–600 K^−1^, which is comparable to value of 451 K^−1^ obtained by *Blanpied et al*. [1995] on Westerly granite. In our case, the slopes of the linear fits strongly depend on the slip history and current sliding velocity. Moreover, the presence of the low‐velocity steps leads to a significant change in (*a‐b*) for velocity steps at higher velocities (Figure 14c), e.g., (*a‐b*) is 0.0137 for the *V =* 1–3 µm/s step in a regular experiment but increases to 0.047 in the slow experiment at *T* = 600°C; the increase in (*a‐b*) is almost completely due to the presence of a large negative second *b* value with a characteristic displacement *d_c_* of 0.3 mm. This was also observed by *Blanpied et al*. [1998].

**Figure 13:**
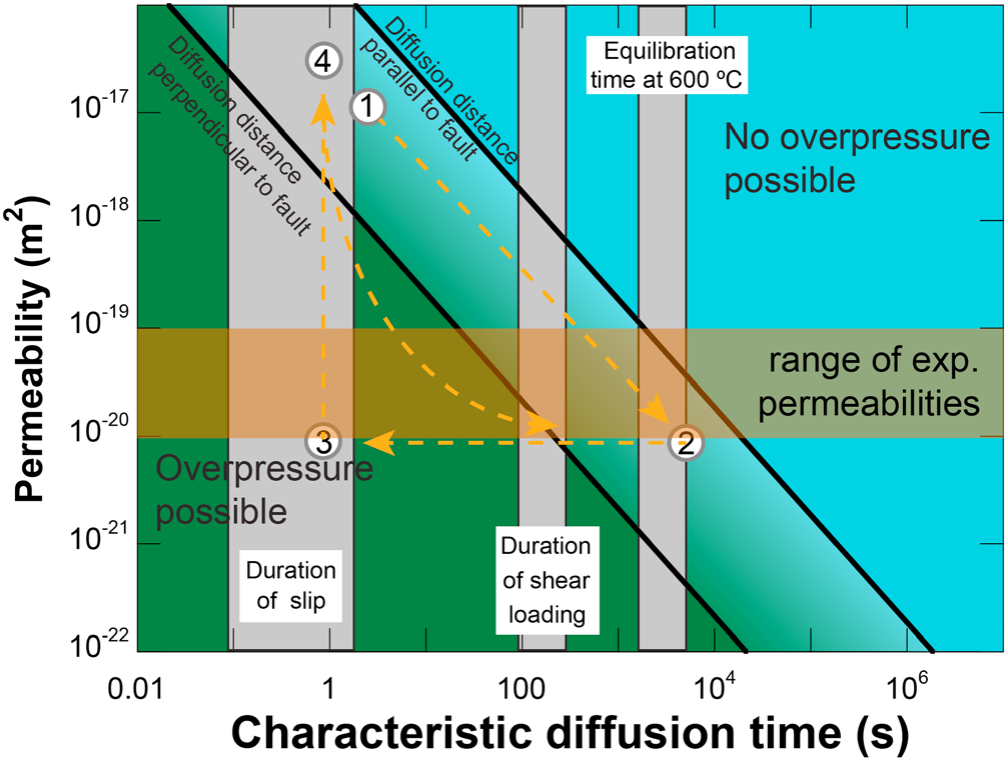
Diffusion times of pore pressure transients calculated for a range of permeabilities. Range of experimental permeabilities is from laboratory measurements on a DFDP‐1B PSZ sample [*Carpenter et al*., 2014] as well as on outcrop samples from fault gouges [*Boulton et al*., 2012]. Our sample probably starts with relatively high permeability at point 1, then moves to point 2 as the applied effective normal stress and temperature reduce its porosity and permeability. Locally, overpressures could develop if the reaction kinetics of fluid‐releasing reactions are fast enough (see main text), eventually causing slip failure (point 3) and a release of pore pressure due to (transiently) increased permeability (point 4).

**Figure 14:**
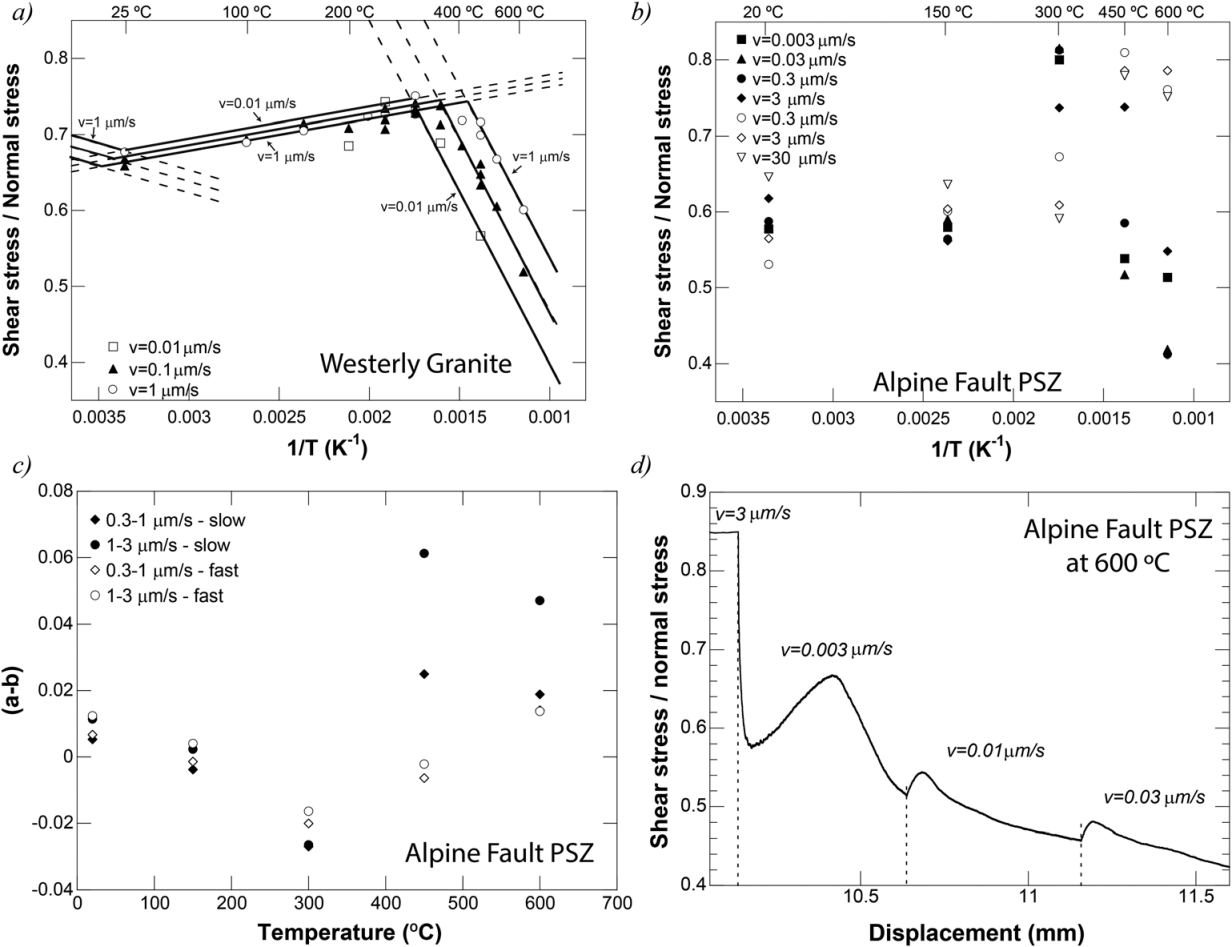
(a) Plot of base friction of Westerly granite as a function of reciprocal absolute temperature. Reproduced from *Blanpied et al*. [1995]. Lines show the fits to the empirical, temperature‐dependent RSF equations. (b) Plot of base friction of sample AF4 as a function of reciprocal absolute temperature for different sliding velocities. Closed symbols are data from the slow experiments, and open symbols are from the regular experiments. Friction was determined at the end of each velocity step. (c) Plot of (*a‐b*) as a function of temperature for the PSZ‐derived sample, AF4, obtained from the regular and slow experiments at 90 MPa effective normal stress. Note the large difference in (*a‐b*) between regular (“fast”) and slow experiments performed at 450 and 600°C. The data point for the *V* = 0.3–1 µm/s step of the “fast” experiment at 600°C is behind the data point of the *V* = 1–3 µm/s step. (d) Plot of the evolution of friction with displacement for the slow velocity steps of experiment u349 at 600°C (sample AF4, derived from the PSZ) showing significant weakening, especially for the lowest velocity.

Our data covering 5 orders of magnitude in sliding velocity show that samples derived from the Alpine Fault remain strong with increasing temperature and show velocity‐weakening behavior, even at temperatures above *T* = 300°C. In fact, unstable stick‐slip events occur at temperatures as high as *T* = 600°C, as discussed in the previous section. The data from the experiments using PSZ‐derived material indicate that for any given temperature, both velocity‐strengthening and velocity‐weakening behaviors can be observed (Figures 7 and 14b), which indicates that the assumption of a single controlling mechanism with constant values for the fitting parameters controlling friction (i.e. (*a‐b*) and *Q*) is invalid. The normal stress‐stepping experiments indicate that sliding is still frictional, i.e. normal‐stress dependent, even at a relatively low sliding velocity of *V =* 0.01 µm/s or a shear strain rate of 10^−5^ s^−1^. Finally, we note that the observed weakening takes considerable displacement to occur (Figure 13).

*Blanpied et al*. [1995, 1998] speculated that the pronounced temperature weakening is the result of localization of slip along a boundary‐parallel shear zone that consists of weaker material, either because it is finer grained or because it contains a through‐going foliation of weak phyllosilicates. In contrast, *Chester and Higgs* [1992] attribute the temperature weakening to the operation of solution‐precipitation processes, aiding distributed granular flow. Yet another explanation could be the activation of dislocation creep in the quartz phase [e.g., *Scholz*, 2002]. We observe localized deformation with uniform extinction only at the higher sliding velocity where friction is high, which contradicts the hypothesis that localized weakness causes weakness at low velocity. Rewriting the flow law for dislocation creep of *Hirth et al*. [2001] for the case of simple shear, we get

(6) $$\dot{\gamma }=3\frac{n+1}{2}Af_{H2O}^{m}\tau^{n}\exp\left(\frac{-Q}{RT}\right)$$

For quartz, *n* = 4, *Q* is the activation energy and is 135 kJ/mol and *m* is 1 [*Hirth et al*., 2001]. For a temperature of 600°C and a fluid pressure of 100 MPa, the fugacity is 64 MPa [*Pitzer and Sterner*, 1994]. Using these values and a strain rate of 10^−5^ s^−1^, we obtain a shear stress of 660 MPa, at least an order of magnitude larger than the shear stress measured. In addition, measured shear stress is linearly dependent on effective normal stress, indicating that sliding is essentially frictional and that the ductility of quartz does not control overall strength.

Intergranular pressure solution (IPS) rates are substantial in quartz under these conditions, especially for very fine grained gouges [e.g., *Niemeijer et al*., 2002]. However, IPS tends to reduce porosity so that shear can only be accommodated if grains also slide past and over each other. Sliding of actively dissolving (and thus cemented) contacts will require high shear stress, and thus, the net result is high friction. However, if sliding can occur over weak phases while intervening clasts deform by diffusive transport, cf. the B‐S model, overall friction can be low. Our results on Alpine Fault materials, in accordance with previous experiments on analogue materials, show that the bulk frictional strength of binary mixtures with a strong, soluble mineral (e.g., halite or quartz) and an aligned weak sheet silicate (e.g., kaolinite or muscovite) can be as low as (or even lower) the strength of the pure weak phase when only 20 wt % of the weak phase is present [*Bos and Spiers*, 2002; *Niemeijer and Spiers*, 2005, 2006; Niemeijer, manuscript in preparation.].

In the Alpine Fault experiments, temperature weakening and associated strong velocity strengthening is only observed in slow experiments after sufficient slip has occurred at a low sliding velocity. The observed variations in both base friction and (*a‐b*) (Figure 14) for the same velocity step in experiments with different sliding histories (i.e., slow versus regular experiments) support our interpretation that foliation development is crucial in enabling frictional‐viscous flow.

## 6 Implications for Alpine Fault Seismogenesis

With reference to determining the area of the Alpine Fault likely to slip seismically, an important question is to what extent the Alpine Fault accommodates deformation interseismically through aseismic creep or slow earthquakes. The absence of creep observed at the surface and the occurrence of microseismicity to depths of 8–10 km suggest that the Alpine Fault is locked at least to these depths [e.g., *Sutherland et al*., 2007; *Boese et al*., 2012; see also *Beavan et al*., 2010; *Lamb and Smith*, 2013]. At the same time, our data suggests that (frictional‐viscous) creep under low stress occurs at a temperature of *T* = 450°C, at least for slip velocities of *V =* 0.003–0.03 µm/s or 94.7 to 947 mm/yr, at least half an order of magnitude faster than the time‐averaged slip rate of the Alpine Fault of 26 mm/yr [*Norris and Cooper*, 2007]. In addition, it is unlikely that the active slip zone width of the Alpine Fault is only 1 mm, as the PSZs drilled in DFDP‐1A and DFDP‐1B are approximately 180–200 mm thick [*Sutherland et al*., 2012; *Toy et al*., 2015]. Considering that the PSZ was most likely active in the brittle part of the crust, the actual width of the deforming zone is probably much larger. If the true slip velocity or strain rate is 2 or 3 orders of magnitude smaller than that simulated in the laboratory, the transitions from velocity strengthening to velocity weakening and back should all shift to lower temperatures. In addition, the displacement‐dependent weakening observed in our experiments at *T* = 450 and *T* = 600°C might occur at much lower temperatures, as also indicated by the B‐S and N‐S models [*Bos and Spiers*, 2002; *Niemeijer and Spiers*, 2007]. Ignoring for the moment the large difference in strain rate between experiment and nature, our results imply that within the framework of RSF, earthquakes are most likely to nucleate at a temperature of 300°C, roughly equivalent to a depth of 6–8 km. The propagation of earthquakes to deeper, hotter levels of the crust is hindered by strong velocity‐strengthening behavior at low strain rate, while at the same time it is promoted by the observed velocity‐weakening behavior at higher strain rates. Large seismic events will be possible if the size of the nucleation patch is large, providing the earthquake with enough energy to propagate through sections of the fault that are interseismically weak and velocity strengthening [e.g., *Boatwright and Cocco*, 1996; *Kaneko et al*., 2010]. To reliably estimate a critical nucleation patch size for the generation of great earthquakes, large‐scale models with realistic, velocity‐dependent RSF parameters should be constructed.

## 7 Conclusions

To investigate the evolution of friction and frictional stability with increasing temperature, we performed a series of hydrothermal frictional sliding experiments on Alpine Fault material, recovered during the Deep Fault Drilling Project (DFDP) phase 1. On the basis of our mechanical and microstructural observations, we can conclude the following:

1. All samples derived from the Alpine Fault are frictionally strong (*μ* > 0.6) under all conditions tested, except when sliding velocity is *V* < 0.3 µm/s and temperature is high (*T* ≥ 450°C).
2. The velocity dependence of friction, expressed as (*a‐b*), shows little variability for the lithologies tested: instead, (*a‐b*) strongly depends on the temperature, sliding velocity, and slip history.
3. All samples show strongly negative values for (*a‐b*) at a temperature of 300°C, also indicated by the occurrence of regular stick slips.
4. Shear stress is linearly dependent on normal stress for all experiments, even at a temperature of *T* = 600°C and a shear strain rate, $\dot{\gamma }$, of ~10^−5^ s^−1^.
5. Preliminary microstructural observations indicate that shear is localized in boundary‐parallel zones of extreme grain size reduction (*d* < 1 µm) when sliding velocity is 10 µm/s. These zones are absent in the sample deformed slowly (*V =* 0.03 µm/s) in which deformation seems to be distributed. In both samples, grain size reduction through cataclasis occurs preferentially in feldspars.
6. We interpret our results to indicate that velocity weakening is the result of the competition between displacement‐dependent granular flow and a thermally activated time‐dependent compactive mechanisms (e.g., pressure solution), which explains the strong dependence of (*a‐b*) on both temperature and sliding velocity.
7. We attribute displacement‐dependent weakening at high temperature and low velocity to the development of and sliding over a frictionally weak foliation, with the accommodation of shear of intervening clasts through time‐dependent deformation. This frictional‐viscous flow mechanism could be an effective weakening mechanism that remains normal stress dependent, even at high temperature.

## Supporting information



Figures S1–S5 and Tables S1 and S2
