# Supplementary material for: Large‐displacement, hydrothermal frictional properties of DFDP‐1 fault rocks, Alpine Fault, New Zealand: Implications for deep rupture propagation
Source: J Geophys Res Solid Earth. 2016 Feb 18;121(2):624–47. doi: 10.1002/2015JB012593 (PMC4994769; doi:10.1002/2015JB012593)
Supplement: Supplementary file 1 — Figures S1–S5 and Tables S1 and S2 [file JGRB-121-624-s001.pdf]

AGU PUBLICATIONS

Journal of Geophysical Research – Solid Earth

Supporting Information for

Large-displacement, hydrothermal frictional properties of DFDP-1 fault rocks, Alpine Fault, New Zealand – implications for deep rupture propagation

A.R. Niemeijer[1], C. Boulton[2], V.G. Toy[3], J. Townend[4] and R. Sutherland[5]

[1] Utrecht University, Faculty of Geosciences, HPT Laboratory, Utrecht, The Netherlands
[2] University of Liverpool, Geology and Geophysics, School of Environmental Sciences, Liverpool, U.K.
[3] University of Otago, Department of Geology, Dunedin, New Zealand
[4] School of Geography, Environment and Earth Sciences, Victoria University of Wellington, New Zealand
[5] GNS Science, Lower Hutt, New Zealand

Contents of this file

Figures S1 to S5
Tables S1 to S2

Additional Supporting Information (Files uploaded separately)

None

Introduction

This supporting information provides additional figures showing the experimental apparatus used, additional experimental data as well as the data use to construct figures 6 & 7 of the main article.

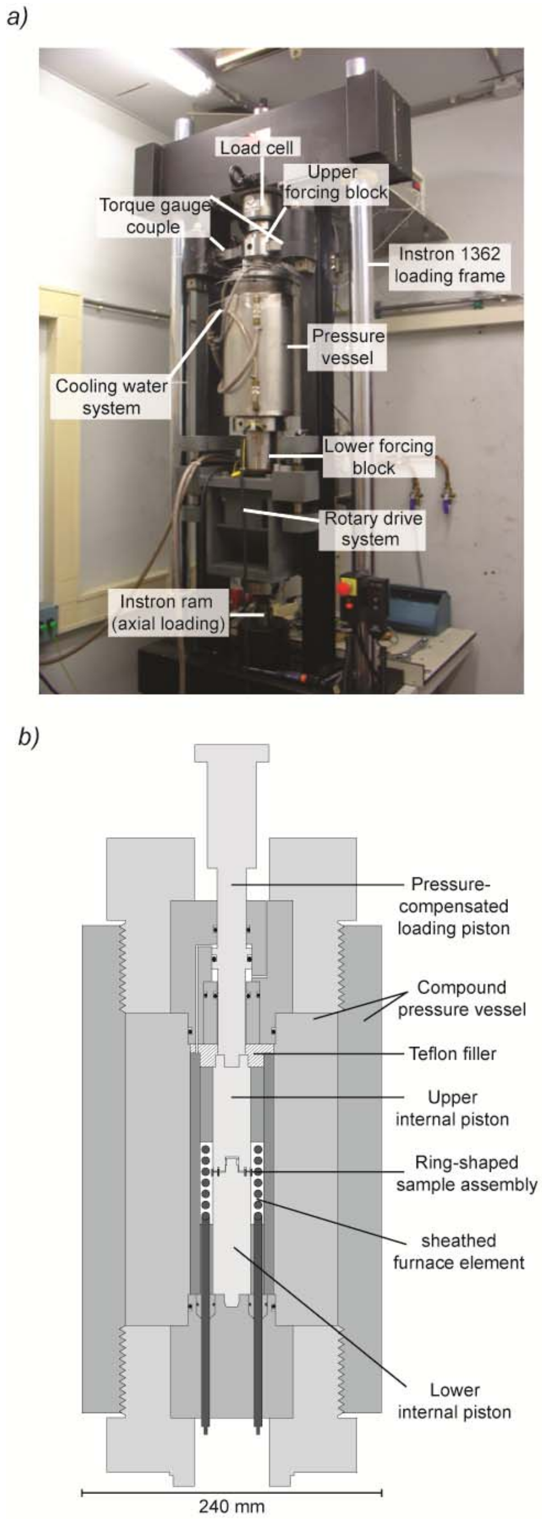

Figure S1.

a) Photograph of the hydrothermal pressure vessel inside the Instron loading frame
b) Schematic drawing of the components of the pressure vessel (after den Hartog et al, 2012).

a) b)

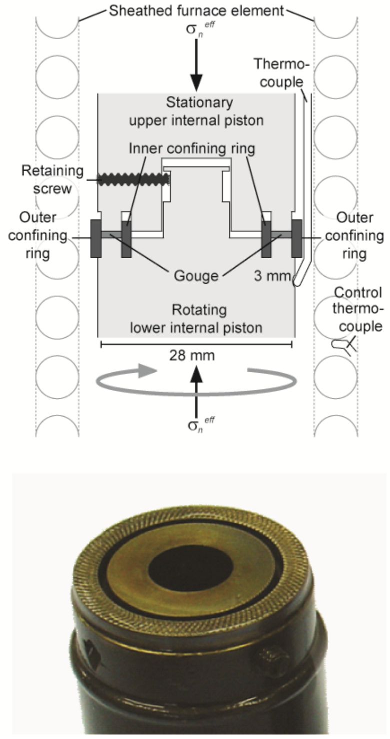

Figure S2.

a) Schematic drawing of the piston assembly (after Niemeijer et al., 2008,den Hartog et al, 2012)

b) Picture of the roughened surface of the top (non-rotating) piston.

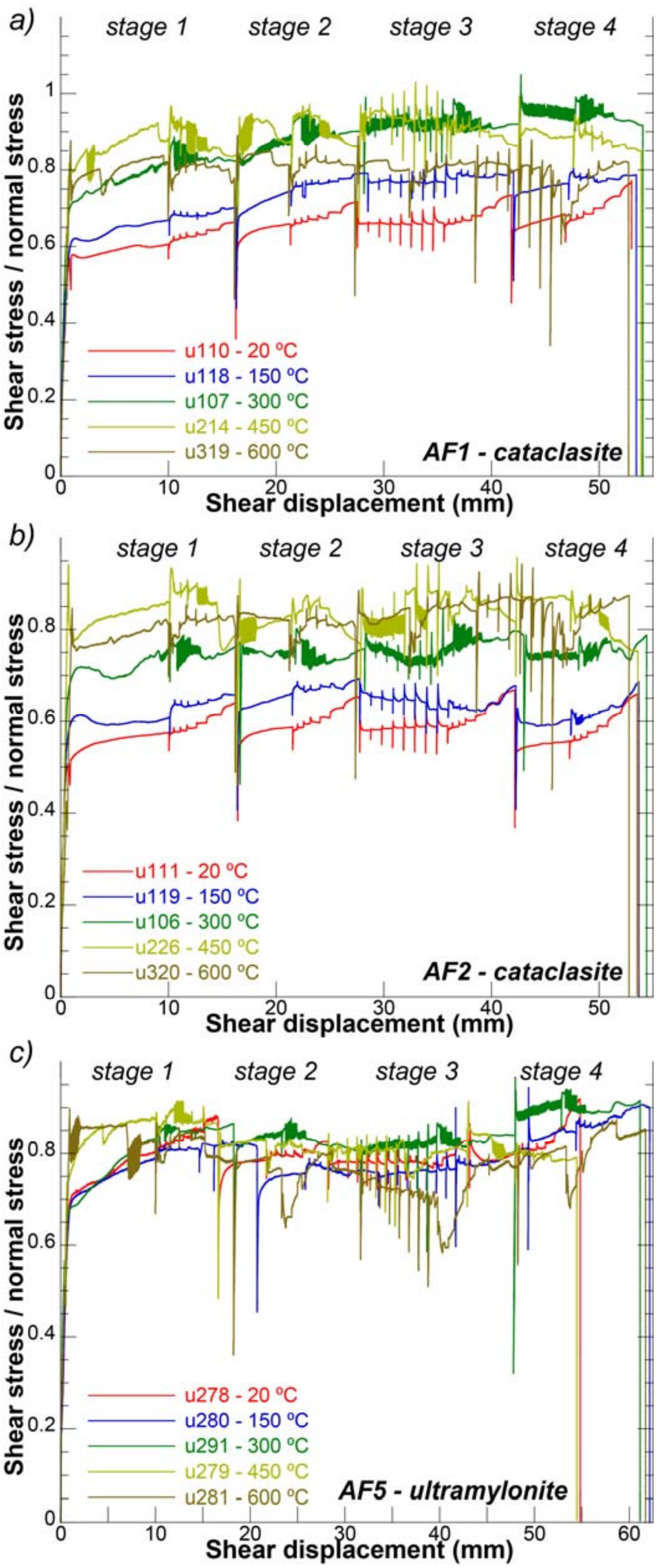

Figure S3.
Evolution of friction with displacement for all samples not shown in the main text.

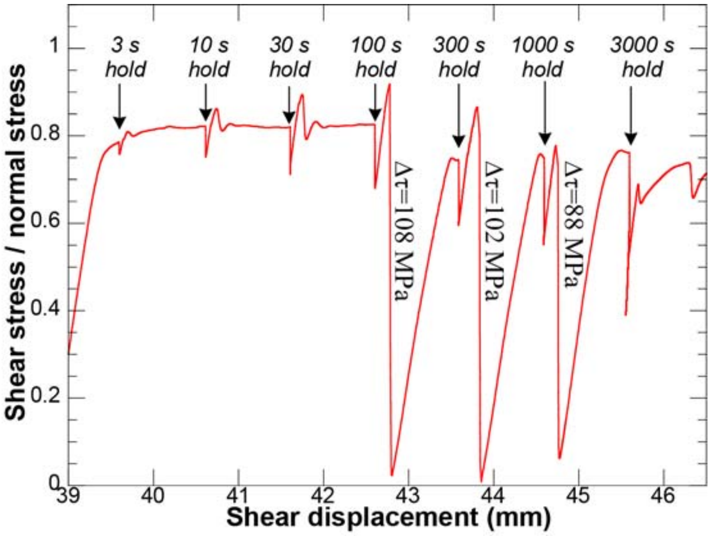

Figure S4.
Plot of friction as a function of shear displacement for experiment u334 (AF4 at 600 ºC), showing the slide-hold-slide sequence. Note the stress drops after holds of 100, 300, 1000 and 3000 seconds, followed by stable sliding.

a)

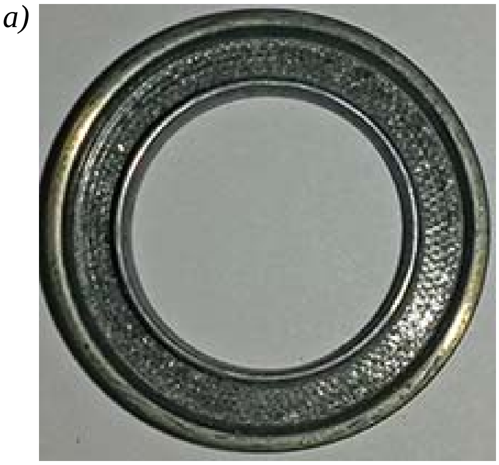

b)

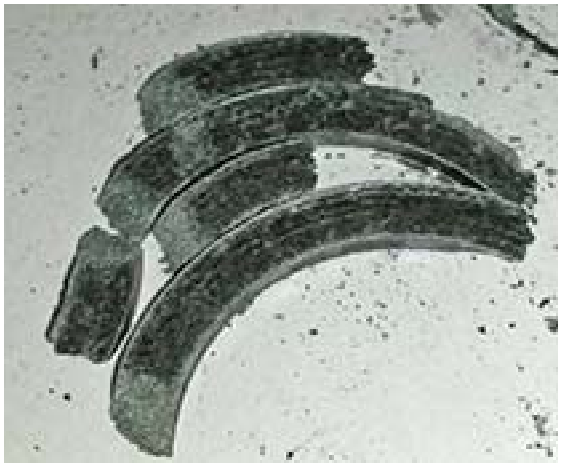

Figure S5.

a) Picture of the sample after experiment u315 showing the intact ring of gouge within the confining rings
b) Picture of the sample shown in a) after removal of the confining rings.

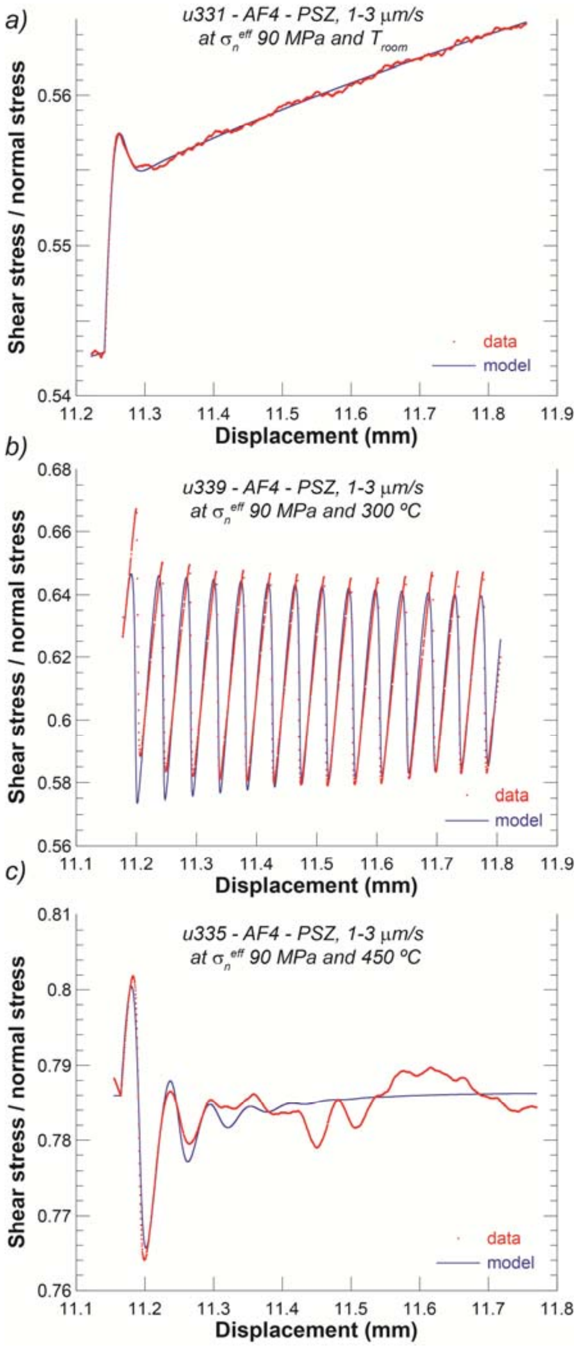

Figure S6.

Examples of model inversions for a velocity step of 1-3 μm/s.

a) T_{room}, a= 0.017585 (stdev= 0.000106), b_1= 0.007634 (0.000102),d_{c1}=7.604 μm (0.132), b_2=-0.00246 (1.77E-05), dc_2= 189.205 μm (4.187), linear trend= 1.38E-05 μm^{-1}, (a-b)=0.012408.

b) T=300 ºC, a= 0.023189 (3E-6), b_1= 0.039548 (2.1E-5),d_{c1}= 5.500195μm (0.007979), linear trend= 0 μm^{-1}, (a-b)= -0.016359.

c) T=450 ºC, a= 0.02484 (0.0003883), b_1= 0.036474 (0.0003),d_{c1}= 7.960276μm (0.192587),), b_2=-0.009452 (0.0005473), dc_2= 41.44665 μm (2.545435),linear trend= 0 μm^{-1}, (a-b)= -0.002182.

Table S1.
(a-b) data corresponding to Figure 6

**AF 1**	*v-step (μm/s)*	*v-step (μm/s)*	*v-step (μm/s)*	*v-step (μm/s)*	*v-step (μm/s)*	*v-step (μm/s)*
T (˚C)	**0.3-1**	**1-3**	**3-10**	**10-30**	**30-100**	**100-300**
**20**	0.000465	0.000995	0.001518	0.0036	0.00697	0.013739
**150**	-0.00266	-0.00445	-0.00188	-0.00397	-0.0026	0.00162
**300**	-0.03946	-0.01591	-0.01593	-0.01992	-0.01252	-0.01012
**450**	-0.00529	-0.00578	-0.02802	-0.03298	-0.0156	-0.01776
**600**	0.036067	0.019816	0.004562	-0.00357	-0.00806	-0.0132
**AF 2**	*v-step (μm/s)*	*v-step (μm/s)*	*v-step (μm/s)*	*v-step (μm/s)*	*v-step (μm/s)*	*v-step (μm/s)*
T (˚C)	**0.3-1**	**1-3**	**3-10**	**10-30**	**30-100**	**100-300**
**25**	0.001024	0.002065	0.002507	0.008024	0.015134	0.013366
**150**	-0.00121	-0.00385	-0.00108	-0.00167	0.000513	0.002196
**300**	-0.02061	-0.02827	-0.01752	-0.00532	-0.00959	-0.00033
**450**	-0.0033	-0.00954	-0.01408	-0.02105	-0.00448	-0.01933
**600**	0.030681	0.027302	0.001963	-0.00761	-0.00732	-0.00762
**AF 5**	*v-step (μm/s)*	*v-step (μm/s)*	*v-step (μm/s)*	*v-step (μm/s)*	*v-step (μm/s)*	*v-step (μm/s)*
T (˚C)	**0.3-1**	**1-3**	**3-10**	**10-30**	**30-100**	**100-300**
**25**	0.001633	0.000157	0.001256	0.004187	0.012433	0.018153
**150**	0.002855	-0.00359	-0.00156	-0.00033	0.000256	0.007032
**300**	-0.0031	-0.00525	-0.00253	-0.00806	-0.00715	-0.00237
**450**	0.019153	0.012829	-0.00874	-0.00975	-0.00259	-0.00543
**600**	0.011772	0.03421	0.021931	0.001792	-0.01348	-0.03599
**AF6**	*v-step (μm/s)*	*v-step (μm/s)*	*v-step (μm/s)*	*v-step (μm/s)*	*v-step (μm/s)*	*v-step (μm/s)*
T (˚C)	**0.3-1**	**1-3**	**3-10**	**10-30**	**30-100**	**100-300**
**25**	0.001345	-0.00287	-0.00022	0.000243	0.001602	0.007305
**150**	-0.00268	-0.00456	-0.00482	-0.0033	0.002976	0.003515
**300**	-0.01237	-0.01744	-0.01615	-0.0048	-0.00515	-0.00308
**450**	-0.02152	-0.02508	-0.02123	-0.02538	-0.01127	-0.00728
**600**	0.014528	0.009727	0.005892	0.009797	-0.00501	-0.00583

Table S2.
(a-b) data corresponding to Figure 7

**AF4**	*v-step* (*μm/s*)	*v-step* (*μm/s*)	*v-step* (*μm/s*)	*v-step* (*μm/s*)	*v-step* (*μm/s*)
T (˚C)	**0.003-0.01**	**0.01-0.03**	**0.03-0.1**	**0.1-0.3**	**0.3-1**
**25**	-0.003735	0.001021	0.000997	0.003947	0.006707
**150**	-0.004858	-0.012287	-0.0134	-0.012755	-0.00142
**300**	0.001458	-0.037982	-0.059043	-0.063866	-0.02003
**450**	0.000804	0.001791	0.015111	0.017841	-0.00638
**600**	-0.011569	0.007362	0.007293	0.020063	0.014052
	**1-3**	**3-10**	**10-30**	**30-100**	**100-300**
**25**	0.012408	0.012974	0.014068	0.011233	0.007222
**150**	-0.00394	0.008012	0.021857	0.022866	0.034731
**300**	-0.01636	-0.00843	-0.01707	-0.00331	-0.0053
**450**	-0.00218	0.001424	-0.0055	-0.01304	-0.01194
**600**	0.013749	0.006799	0.012919	-0.0084	-0.00564
